# Systematic analysis and expression of *Gossypium* 2ODD superfamily highlight the roles of *GhLDOXs* responding to alkali and other abiotic stress in cotton

**DOI:** 10.1186/s12870-023-04133-x

**Published:** 2023-03-04

**Authors:** Tiantian Jiang, Aihua Cui, Yupeng Cui, Ruifeng Cui, Mingge Han, Yuexin Zhang, Yapeng Fan, Hui Huang, Xixian Feng, Yuqian Lei, Xiaoyu Liu, Kesong Ni, Hong Zhang, Nan Xu, Jing Wang, Liangqing Sun, Cun Rui, Junjuan Wang, Shuai Wang, Xiugui Chen, Xuke Lu, Delong Wang, Lixue Guo, Lanjie Zhao, Fushun Hao, Wuwei Ye

**Affiliations:** 1 State Key Laboratory of Cotton Biology / School of Life Sciences, Henan University, Kaifeng, 475004 Henan China; 2 Research Base, Anyang Institute of Technology, State Key Laboratory of Cotton Biology / Institute of Cotton Research of Chinese Academy of Agricultural Sciences, Anyang, 455000 Henan China; 3 Cotton Research Institute of Jiangxi Province, Jiujiang, 332105 Jiangxi China

**Keywords:** Leucocyanidin dioxygenase, Gene family, Phylogenetic analysis, Structural analysis, Cotton

## Abstract

**Background:**

2-oxoglutarate-dependent dioxygenase (2ODD) is the second largest family of oxidases involved in various oxygenation/hydroxylation reactions in plants. Many members in the family regulate gene transcription, nucleic acid modification/repair and secondary metabolic synthesis. The 2ODD family genes also function in the formation of abundant flavonoids during anthocyanin synthesis, thereby modulating plant development and response to diverse stresses.

**Results:**

Totally, 379, 336, 205, and 204 2ODD genes were identified in *G. barbadense* (Gb), *G. hirsutum* (Gh), *G. arboreum* (Ga), and *G. raimondii* (Gb), respectively. The 336 2ODDs in *G. hirsutum* were divided into 15 subfamilies according to their putative functions. The structural features and functions of the 2ODD members in the same subfamily were similar and evolutionarily conserved. Tandem duplications and segmental duplications served essential roles in the large-scale expansion of the cotton 2ODD family. *Ka/Ks* values for most of the gene pairs were less than 1, indicating that 2ODD genes undergo strong purifying selection during evolution. Gh2ODDs might act in cotton responses to different abiotic stresses. *GhLDOX3* and *GhLDOX7*, two members of the GhLDOX subfamily from *Gh2ODDs*, were significantly down-regulated in transcription under alkaline stress. Moreover, the expression of *GhLDOX3* in leaves was significantly higher than that in other tissues. These results will provide valuable information for further understanding the evolution mechanisms and functions of the cotton 2ODD genes in the future.

**Conclusions:**

Genome-wide identification, structure, and evolution and expression analysis of 2ODD genes in *Gossypium* were carried out. The 2ODDs were highly conserved during evolutionary. Most Gh2ODDs were involved in the regulation of cotton responses to multiple abiotic stresses including salt, drought, hot, cold and alkali.

**Supplementary Information:**

The online version contains supplementary material available at 10.1186/s12870-023-04133-x.

## Background

Approximately 7% of the world’s land (more than 900 million hectares) is threatened by saline-alkalization stress. There are no effective measures to control soil salinization currently [1]. Thus, soil saline-alkalization has become a major limiting factor for crop production in global agriculture [2]. The effects of saline-alkalization on plants include the effects of both salt and alkali stress. Alkali stress is mainly induced by NaHCO_3_ and Na_2_CO_3_ while salt stress results from NaCl, Na_2_SO_4_ and other neutral salts. Compared with salinity, alkali stress causes greater damage to plants [3]. Although cotton plants are salt-tolerant, their growth and development can be severely affected by salt and alkali stresses [4].

2ODDs are non-heme proteins belonging to the second largest oxidase family in plants. They regulate various metabolic activities during growth and development of plants. For instance, it exerts effects in diverse primary metabolism processes including the synthesis and catabolism of gibberellins (GAs), ethylene biosynthesis, and catabolism of auxin and salicylic acid (SA). They also modulate secondary metabolisms, such as the biosynthesis and/or metabolism of benzylisoquinoline alkaloids (BIAs), glucosinolates, tropane alkaloids (TAs), monoterpene indole alkaloids (MIAs), benzoxazinoids, coumarins, mugineic acid, steroid glycoalkaloids (SGs) and flavonoid [5].

Anthocyanins are a class of flavonoids that participate in a variety of biological processes. They are widely distributed in plant leaves, flowers, stems, fruits and other organs, making these organs’ different colors. There are hundreds of naturally occurring anthocyanins. Six of them are common ones, cyanidin, delphinidin, petunidin, peonidin, pelargonidin, and malvidin [6]. In the edible parts of plants, cyanidin accounts for about 50%, pelargonidin, peonidin and malvidin acount for 12%, and petunidin and delphinidin account for 7% of the total contents of anthocyanins, respectively [7].

When plants are subjected to various biotic and abiotic stresses such as pathogens, low temperature, drought, UV and phosphate deficiency, the anthocyanin in tissues accumulates to resist the stresses [8–10]. Anthocyanin has antioxidant activity, and can effectively scavenge reactive oxygen species, reducing cell damage caused by cell membrane swelling when plants are exposed to different stresses [11]. Furthermore, as a pure natural antioxidant, anthocyanin is safe and non-toxic, and can inhibit cancer, cardiovascular disease and other diseases to a certain extent in human beings [12, 13].

The anthocyanin biosynthesis is a branch of the flavonoid and phenylpropane biosynthesis [6]. It begins with the formation of chalcone catalyze by chalcone synthase. Then, a series of key enzymes including chalcone isomerase (CHI), flavanone-3-hydroxylase (F3H), dihydroflavonol-4-reductase (DFR), flavonol synthase (FLS), colorless leucocyanidin dioxygenase/anthocyanin synthase (LDOX/ANS) participate in the biosynthetic reactions, leading to the production of brick red/orange pelargonidin, red/pink cyanidin and blue/purple delphinidin [14]. Finally, under the action of glycosyltransferase (GTs), unstable anthocyanin forms a glycosidic bond with one or more glucose, rhamnose, galactose, xylose and arabinose and converts into stable anthocyanin [15].

LDOXs are 2-ketoglutarate-dependent dioxygenases belonging to the 2ODDs. They can convert colorless-anthocyanin into unmodified colored anthocyanin. Studies have shown that Fe^2+^, α-ketoglutarate, dioxygen and ascorbate can bind to LDOXs, causing the oxidative decarboxylation of LDOXs. Thus, succinate and carbon dioxide generate. The intermediate products of these reaction are dehydrated to form C-2, C-3 enol or C-3, C-4 enol, wherein C-2 and C-3 enol forms colored anthocyanin, and C-3 and C-4 enols form dihydroflavonols [16, 17].

Evidence indicates that some genes involved in the anthocyanin synthesis can enhance stress resistance by increasing the accumulation of anthocyanin and flavonoids in plants under stresses [18]. In rice, overexpression of *OsCHI2* causes significant increases in the expression levels of genes implicated in flavonoid biosynthetic pathway, better growth status and higher survival rates [19]. Similarly, in Arabidopsis, *PnF3H*, *AtDFR* and *RtLDOX2* genes can confer stress resistance to plants by raising anthocyanin and flavonol contents [20–23]. Also, in tobacco, overexpression of the anthocyanin synthesis-related gene *AvFLS* improves salt tolerance by increasing the total flavonoid contents [24].

LDOXs can convert leucocyanidin into colored anthocyanin, which is the first colored compound in the anthocyanin metabolic pathway. Evidence indicates that suppressing the expression of the *LDOX* gene causes flowers lighter or even whiter. These are very intuitive phenotypes. After knockdown of the expression of the *ANS* gene of *Torenia fournieri*, the flower color changes from the blue to the white. In rice, overexpressing the *ANS* gene results in a clear enhancement of the anthocyanin content, and the seed coat becomes purple-red. ANS is a LDOX protein [25]. Consistently, the expression level of *LDOX* gene in red petals is considerably higher than that in white petals of *Magnolia sprengeri Pampan* [26]. Additionally researchers investigated the expression of *LDOX* gene in the peel of 8 different grape varieties, and found that the transcript abundances of *LDOX* are markedly higher in the peel of the red variety, but lower in the peel of the white variety [27]. To date, the involvement of *LDOXs/ANSs* in stress resistance has been reported in some plant species. However, whether LDOXs serve roles in cotton is unknown. Therefore, it is of great significance to study the functions of cotton LDOXs in stress resistance.

In this study, 336 2ODDs in *G. hirsutum* were identified. They were divided into 15 subfamilies. The phylogenetic analyses showed that 2ODD family members were relatively conservative during evolution. Tandem duplications and segmental duplications served important roles in the large-scale expansion of 2ODDs. *GhLDOX3*, a 2ODD family gene, was isolated and characterized. VIGS plants with silenced *GhLDOX3* were insensitive to Na_2_CO_3_ stress. These data will provide new insights into the evolutionary history and functions of cotton 2ODDs in metabolite biosynthesis and stress responses.

## Results

### Identification of 2ODD proteins

In order to obtain the *2ODD* family genes in four *Gossypium* species, *G*. *arboreum*, *G. raimondii*, *G. hirsutum* and *G. barbadense*, the 2ODD Hidden Markov model in Pfam was used to search the genomes of these species in the HMMER software. The candidate proteins were then verified by the NCBI-CDD and Pfam tools as 2ODDs contains DIOX_N domain and 2OG-FeII_Oxy domains. Therefore, the candidates with incomplete domains of DIOX_N and 2OG-FeII_Oxy were deleted manually. In total, 1124 *2ODD* genes were identified, 379 from tetraploid *G. barbadense*, 336 from tetraploid *G. hirsutum*, 205 from diploid *G. arboreum*, and 204 from diploid *G. raimondii*. These genes were renamed according to their locations on the chromosomes (Table S1).

Allotetraploid *G. hirsutum* was originated from the genomic hybridization between an ancestral *G. arboreum* with an A genome and *G. raimondii* with a D genome [28]. It is a good model for studying the evolution and origin of polyploidy plants. Accordlingly, we focused on the *2ODD* genes in *G. hirsutum*, and compared these genes with their homologs in other three *Gossypium* species. The physicochemical properties of these genes or their encoded proteins were then analyzed and predicted, including transcript length, CDS length, GC content of CDS, exon number, average exon length, average intron length, protein length, protein molecular weight, isoelectric spot, protein hydrophobicity and subcellular localization (Table S2). The results showed that the smallest molecular weight of the Gh2ODDs in *G. hirsutum* was 5.753 kDa (GhF6H14), the largest was 59.301 kDa (GhH6H8). The number of amino acid of Gh2ODDs ranged from 52 (GhF6H14) to 522 (GhH6H8), and the isoelectric points of these proteins were 4.266 to 6.248. Gh2ODDs’ hydrophobicity coefficients were from positive 1.79% to negative 98.21%, indicating that most of the proteins in this family are hydrophilic. It was predicted that 168 *Gh2ODDs* were localized in the cytoplasm, 74 *Gh2ODDs* in the nucleus, 47 *Gh2ODDs* in the chloroplast, 25 *Gh2ODDs* in the cytoskeleton, 8 *Gh2ODDs* in the mitochondrium, 5 *Gh2ODDs* in the peroxisome, 3 *Gh2ODDs* in the extracellular matrix, 3 *Gh2ODDs* in the endoplasmic reticulum, 1 *Gh2ODDs* in the vacuole, and 1 *Gh2ODDs* in the plasma membrane.

### Phylogenetic analysis

**Fig. 1:**
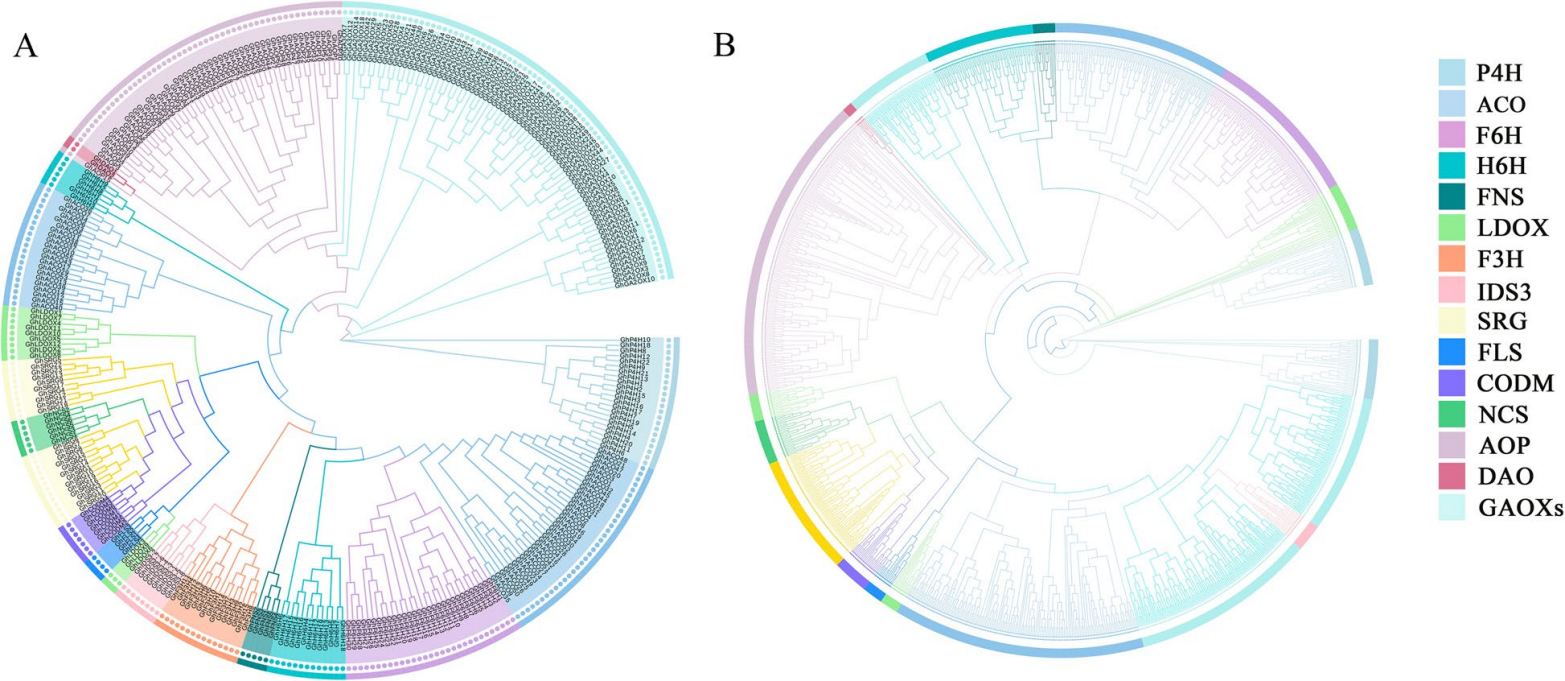
Two rootless phylogenetic trees for 2ODDs. **A** A phylogenetic tree of 336 2ODDs in *G. hirsutum* by Neighbor-Joining (NJ) method. **B** A phylogenetic tree for the 1124 identified 2ODDs from four *Gossypium* species by Maximum Likelihood (ML) method. Boxes with varying color represent different clades of 2ODDs

In order to study the evolutionary relationship of the 2ODD family members, a rootless phylogenetic tree was constructed using the 336 2ODD genes of *G. hirsutum* (Fig. 1A). The 2ODD proteins contained the highly conserved DIOX_N domain and 2OG-FeII_Oxy domain. With the recombination, duplication and divergence of some protein sequence, the functions of 2ODDs might become more and more diverse. According to the protein structure and putative functions, the 336 2ODD genes of *G. hirsutum* can be divided into 15 subfamilies, named respectively GhP4H, GhACO, GhF6H, GhH6H, GhFNS, GhLDOX, GhF3H, GhIDS3, GhSRG, GhFLS, GhCODM, GhNCS, GhAOP, GhDAO and GhGAOX. Among them, GhGAOX has 76 members, being the most, and the GhDAO are the least, with only 2 members. The rootless phylogenetic tree of 1124 2ODD proteins among four *Gossypium* species (336 in *G. hirsutum*, 379 in *G. barbadense*, 205 in *G. arboreum*, 204 in *G. raimondii*) was constructed (Fig. 1B). The four *Gossypium* species were likewise divided into 15 subfamilies. The comparison shows that the ratio of the number of *2ODD* genes in the two diploid cottons is close to 1:1, and the ratio of the number of *2ODD* genes in the A subgenome to the D subgenome in the two tetraploid cottons is close to 1:1. But it is less than the number of *2ODD* genes in the two diploid cotton genomes. This may be due to gene loss during the hybridization of two diploid *Gossypium* to form heterotetraploid *Gossypium.*

### Organization of *2ODD* genes on chromosomes of four *Gossypium* species

**Fig. 2:**
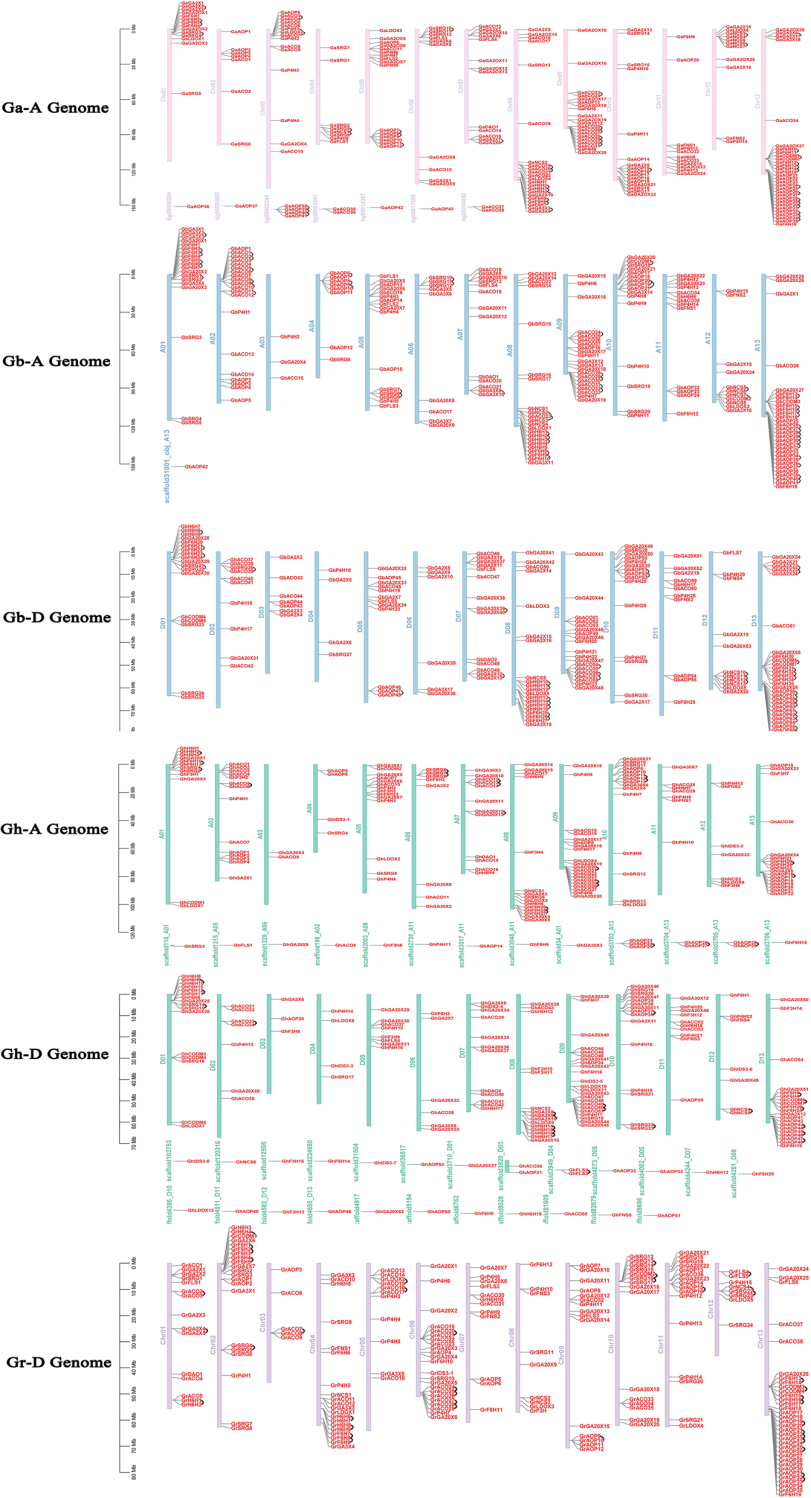
Chromosomal positions of *2ODDs* from four *Gossypium* species. Gene IDs are shown on the right side. The vertical bar on the left side represents the position of the genes and length of chromosomes. Black lines indicate tandem duplication gene pairs

To further study the genetic differentiation and gene duplication events of the *2ODD* genes in four *Gossypium* species, their chromosome maps were constructed (Fig. 2). Among the 1124 *2ODDs*, 1071 were assigned to their specific chromosomes, and the remaining 53 were assigned to unlabeled chromosomes. We therefore did not analyzed the 53 *2ODDs* in detail. The vast majority of the 1071 *2ODDs* were located at the ends of chromosomes (near telomeric region), and a few of them were distributed in non-telomeric and centromeric regions of chromosomes. Among the 336 *2ODDs* of *G. hirsutum*, 164 and 172 were located on the chromosomes of the At subgenome (GhAt) and Dt subgenome (GhDt), respectively; 17 and 25 were positioned on the scaffolds of GhAt and GhDt, respectively. The 13th and 9th chromosomes harbored 20 and 17 *2ODDs*, respectively, while the 3rd and 4th chromosomes had only 2 and 4 *2ODDs*, respectively. In addition, the number of *Gh2ODDs* on 13 chromosomes in GhAt and GhDt was the same, indicating that these genes are relatively conservative during evolution.

In *G. barbadense*, 202 of the 379 *2ODDs* were located on the chromosomes of the At subgenome (GbAt), and the remaining 177 *Gb2ODDs* were placed on the chromosomes of the Dt subgenome (GbDt). The thirteenth chromosomes in both GbAt and GhDt contained 30 *Gb2ODDs*. By contrast, the third chromosome of GbAt had only three *Gb2ODDs*, and the fourth chromosome of GhDt harbored only four *Gb2ODDs* (Fig. 2). The number of *Gb2ODDs* on the homologous chromosomes of A and D subgenomes in *G. barbadense* was largely different, suggesting that loss and addition events of *Gb2ODDs* occur during evolution.

There were 205 *Ga2ODDs* in *G. arboreum* and 204 *Gr2ODDs* in *G. raimondii*. These genes were evenly distributed on 13 chromosomes in the two species. Among the 205 *Ga2ODDs*, 12 were not fixed on specific chromosomes. We found that the majority of *2ODD* genes in the four *Gossypium* species were evenly distributed on their homologous chromosomes, but some genes were not during evolution. This suggests that the genome of the two diploid speicies were directly inherited although the genomes of the allotetraploid AD are derived from the hybridization of the two diploid A and D genomes. However, during the long-term evolutionary process, gene loss and chromosome fusion events also occurred in the *Gossypium* genomes, and evolutionary asymmetry emerged (Table 1).

**Table 1:** Distributions of *2ODD* genes on different genomes and sub genomes of four *Gossypium* species (Ga, Gr, Gh, Gb)

Chr.No	Ga	Gh-At	Gb-At	Gr	Gh-Dt	Gb-Dt	Total
Chr. 1	12	12	19	15	16	18	92
Chr. 2	7	14	20	21	7	10	79
Chr. 3	15	2	3	5	3	7	35
Chr. 4	8	4	8	20	4	4	48
Chr. 5	16	14	16	11	8	12	77
Chr. 6	10	8	9	23	6	6	62
Chr. 7	13	12	13	12	12	14	76
Chr. 8	22	15	22	9	15	20	103
Chr. 9	20	20	21	15	23	19	118
Chr. 10	16	15	16	15	15	15	92
Chr. 11	11	7	13	16	10	11	68
Chr. 12	11	7	11	8	7	11	55
Chr. 13	30	17	30	34	20	30	161
Scaffolds	14	17	1	0	26	0	58
Total	205	164	202	204	172	177	1124

### Duplication and collinearity analysis of *2ODD* genes

**Fig. 3:**
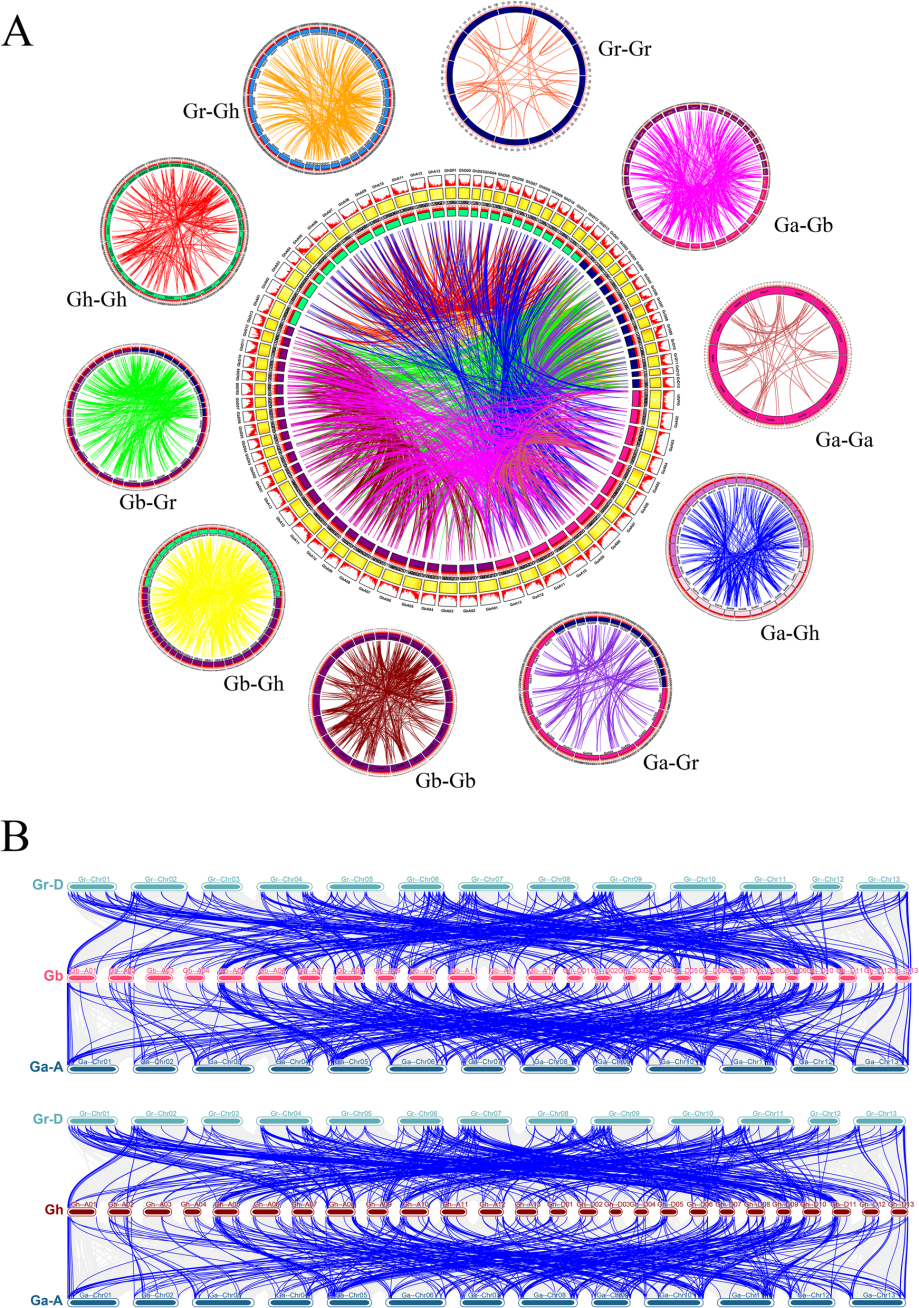
Collinearity relationships of *2ODDs* in *Gossypium*. **A** The collinearity relationship of repeated gene pairs of *2ODDs* among *G. hirsutum, G. barbadense, G. arboreum* and* G. raimondii.* The chromosomal lines in different colors indicate the collinearity area around the *2ODDs*. **B** Collinearity relationships of *Gh2ODDs* and *Gb2ODDs* with their homologs in the ancestor species through multiple synteny plots. Dense grey lines in the background reveal collinear blocks, while blue lines represent syntenic 2ODD gene pairs

The evolutionary relationship and collinearity of 2ODDs in two diploid ancestors *G. arboreum* (AA) and *G. raimondii* (DD) and two tetraploid progenies *G. hirsutum* (AtAtDtDt) and *G. barbadense* (AtAtDtDt) were analyzed. The results showed that the subgenomes Ga-Ga, Ga-Gb, Ga-Gr, Ga-Gh, Gb-Gb, Gb-Gr, Gb-Gh, Gr-Gr and Gr-Gh were collinear. There were 3631 orthologous/paralogous gene pairs in Gh-Gh, 597 pairs of segmental duplications, 219 pairs of tandem duplications, and 2815 pairs of whole genome duplication (WGD). The latter enabled a large-scale extension of the 2ODD superfamily in *Gossypium*. *G. barbadense* had the most tandem duplications (90 pairs), followed by *G. arboreum* (48 pairs), *G. hirsutum* (42 pairs), and *G. raimondii* (39 pairs) (Fig. 3A). It was found that a number of *2ODDs* in diploid A and D genome were orthologous genes in the tetraploid At and Dt subgenomes (Fig. 3B). Our findings indicate that many gene loci are highly conserved between At subgenomes and A genomes as well as Dt subgenomes and D genomes (Fig. 3B). In addition, it was seen that Ga-A genome had more homologous gene pairs with the subgenomes of Gh-At and Gb-At on chromosomes 5, 8, 9, 10 and 11 than other chromosomes; and Gr-D genome had more homologous gene pairs with Gh-Dt and Gb-Dt subgenomes on chromosomes 4, 6, 7, 9 and 11 than other chromosomes (Fig. 3B).

### Selection pressure analysis of 2ODDs among four *Gossypium* species

**Fig. 4:**
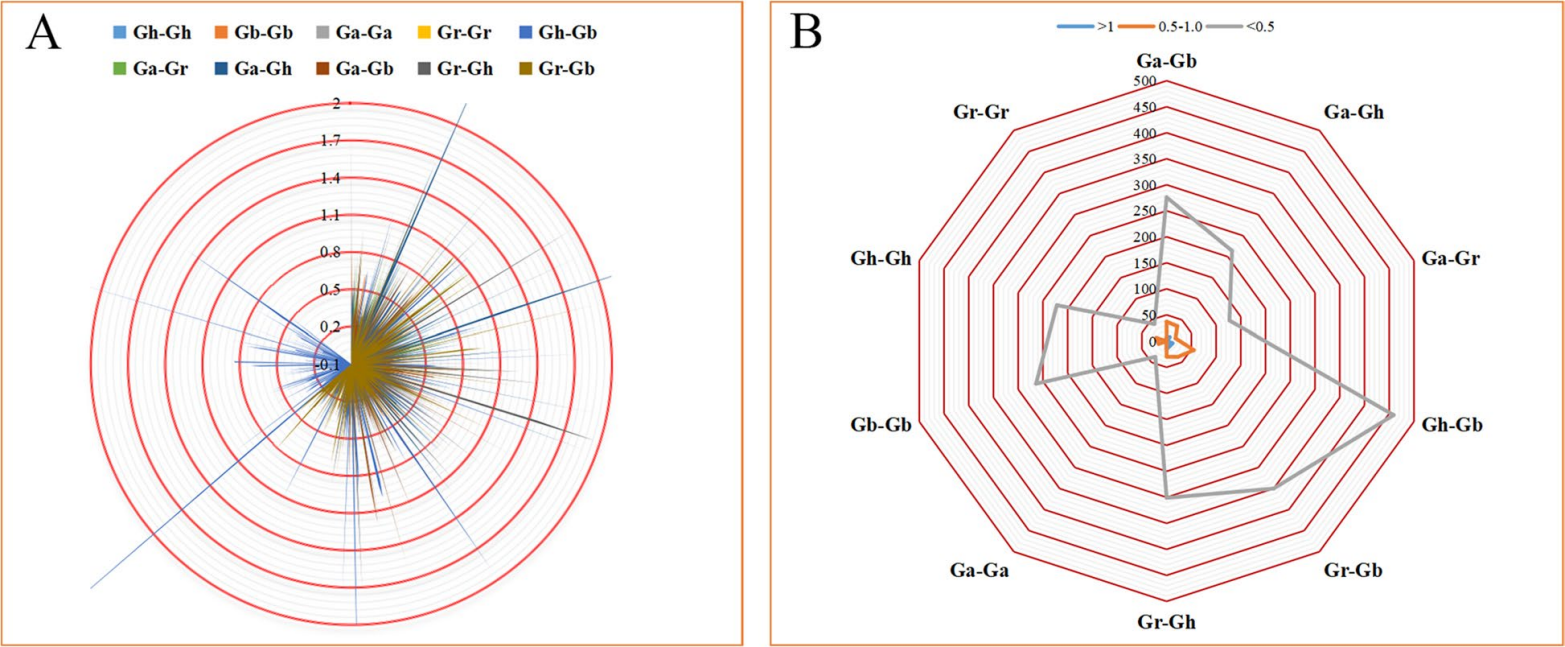
Selection pressure based on non-synonymous to synonymous ratio (*Ka/Ks*) for *2ODDs*. **A** The density of duplicated gene pairs in various ranges of *Ka/Ks.* Different colors mean different combinations of gene pairs among four *Gossypium* species. **B** Prediction of a number of duplicated gene pairs of different combinations from four *Gossypium* species. The blue, orange and grey colors show the different selection pressure

In order to determine the effects of Darwinian positive selection and selection pressure on the evolution of *2ODD*s, their non-synonymous substitution rate (*Ka*) and the synonymous substitution rate (*Ks*) of 2595 pairs of homologous genes were calculated between genomes and within genomes/subgenomes pairs of four *Gossypium* species. The *Ka/Ks* ratio, which reflects the selection pressure of homologous genes, was analyzed. The results showed that there were 57 pairs of homologous genes (accounting for 2.3%) with a *Ka/Ks* ratio greater than 1, indicating that these gene pairs were subjected to positive selection during the evolutionary process and occurred under strong artificial selection pressure. Favorable variation is evidence of adaptive evolution of proteins. The *Ka / Ks* ratio for 2538 pairs of homologous genes (97.7%) was less than 1. Of these pairs, 2290 pairs had a *Ka / Ks* ratio lower than 0.5, and 248 pairs had the ratio between 0.5 and 1 (Fig. 4), indicating that these genes are affected by purification selection. Next, the average separation time between the two diploid species as well as between the two tetraploid species above was evaluated and the average *Ka/Ks* value was assessed. The average separation time of *G. arboreum* and *G. raimondii* was the earliest, and the average ratio of *Ka / Ks* was the smallest. The average separation time of *G. barbadense* and *G. hirsutum* was shorter than that of *G. arboreum* and *G. raimondii*, but their average *Ka / Ks* ratio was greater than that of *G. arboreum* and *G. raimondii* (Table S3). These results indicate that *G. arboreum* and *G. raimondii* separated first, but are relatively conserved during evolution, and then *G. barbadense* and *G. hirsutum* separated after a long time, but undergo relatively rapid and complex evolution.

### Structure and conserved motifs of Gh2ODDs

**Fig. 5:**
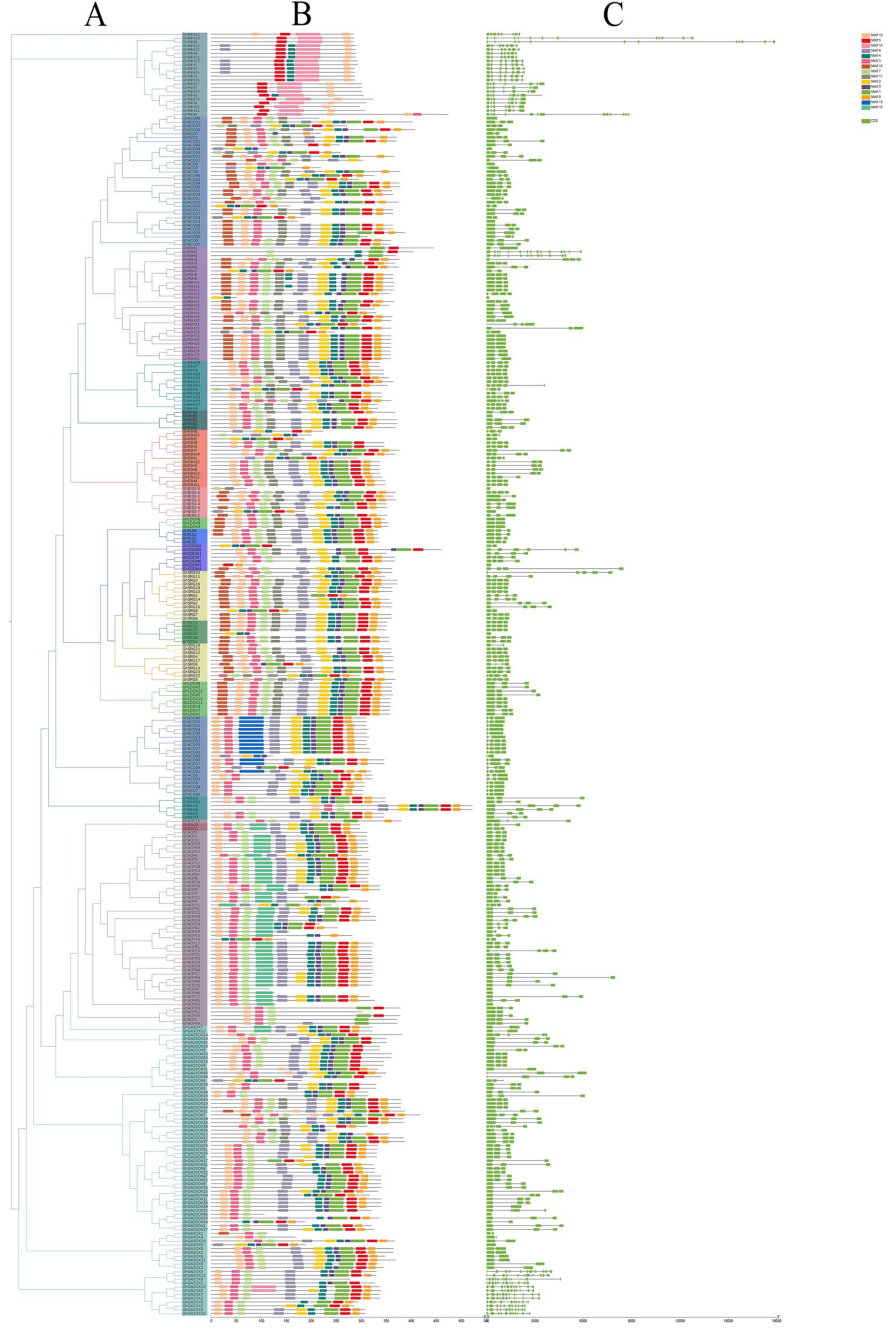
Conserved protein motifs of Gh2ODDs along the phylogenic tree and subfamily classification patterns. **A** Phylogenetic tree of Gh2ODDs. **B** Conserved motifs of Gh2ODDs. Boxes with varying color represent different motifs. **C** Gene structure of *Gh2ODDs*. Green boxes indicate exons, and black lines show introns

To further understand the structural evolution of *Gh2ODDs*, the protein sequences of Gh2ODDs were used to construct a phylogenetic tree. We found that they were classified according to the tree topology of the evolutionary tree (Fig. 5A). We also investigated conserved protein motifs of the Gh2ODD members in each subfamily, and found that a total of 15 motifs (motif 1-motif 15) were detected in Gh2ODDs (Fig. 5B). Most members of the same subfamily, especially closely related members had the same motif composition. There are 1–13 motifs in each Gh2ODD proteins, and most members had the same motif distribution. This means they have similar functions at the protein level. Most Gh2ODD proteins were observed to contain motifs 1, 3, 4, 5, and 10 in common. The number of motifs in the GhP4H subfamily was relatively small. They shared motifs 5, 10, and 14. Some members had motifs 4 and 6. Motif 14 was unique to the GhP4H subfamily. Most members of the other six subfamilies had more than 10 motifs, and a few members had lost part of their functions during evolution. Motif 12 was unique to the GhACO, GhF6H, GhF6H, GhIDS3, GhLDOX, GhFLS, GhSRG and GhNCS subfamilies, 15 motifs were exclusive to some GhACO members, and 13 motifs were unique to the GhAOP, GhDAO subfamilies. The conserved motifs of each subfamily with their own unique arrangement may reflect the functional specificity of each subfamily.

The *GhF6H2* had 15 exons, being the largest number, whereas some had only 1, being the least number. It is interesting that the exon/intron arrangement of closely related genes like GhLDOX members was more similar, but their exon/intron length was clearly different. Among the 336 *Gh2ODDs*, 16 were intron-less, accounting for 4.8% of the total number of genes, and 55 contained one intron, accounting for 16% of the total number of genes (Fig. 5C, Table S2). Fewer introns in these genes suggest that alternative splicing events of these *Gh2ODDs* may not happen during evolution. We found that the *2ODDs* in 7 subfamilies (GhP4H, GhH6H, GhLDOX, GhF3Hs, GhSRG, GhFLS and GhDAO) contained at least one intron, indicating that those subfamilies are evolutionarily conservative. There were only 1 to 2 introns in 12 members of the GhLDOX subfamily. The other 8 subfamilies (GhACO, GhFNS, GhIDS3, GhCODM, GhNCS, GhAOP, GhGAOX and GhF6H subfamilies) contained intronless members (Fig. 5C), indicating these 2ODD members may be involved in response to alkaline stress in cotton. It is worth noting that the genetic structure of closely related genes was more similar, but the length of exons/introns was not necessarily the same.

### Analyses of *cis*-regulatory elements and expression profiles of *Gh2ODDs*

**Fig. 6:**
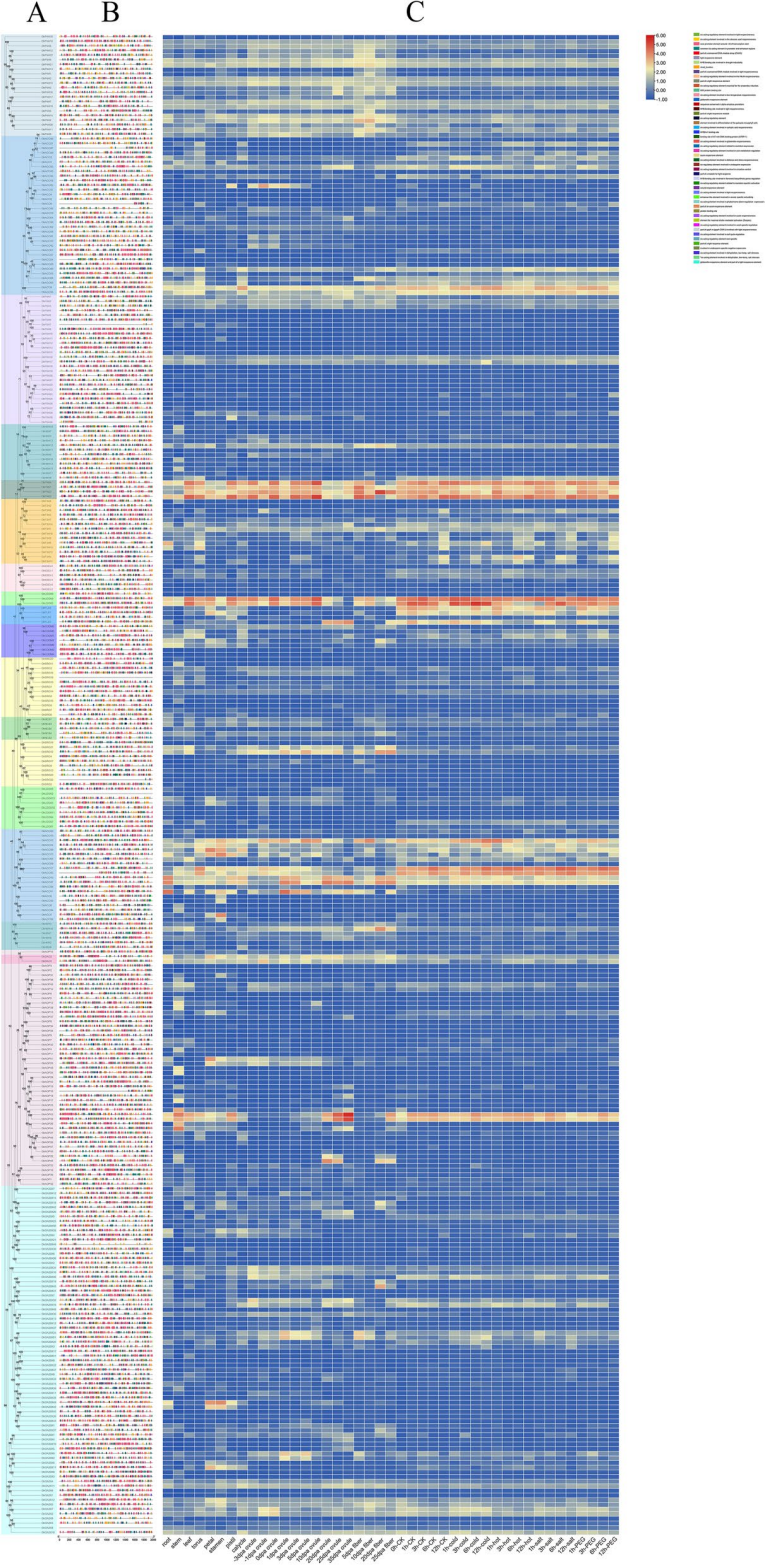
Analysis of *cis* elements and expression profiles of *Gh2ODDs.* **A** Phylogenetic tree of *Gh2ODD* genes. **B** *Cis* regulatory elements present in promoter regions of *Gh2ODDs*. Bars in diverse colors indicate different *cis*-acting elements. **C** Differentially expressed *Gh2ODDs* under cold, hot, salt or drought stress, as well as tissues expression patterns of *Gh2ODDs* at various growth stages. The expression level from lower to high are represented by red bar to blue one

*Cis*-regulatory elements (CREs) are non-coding DNAs in genes containing binding sites for transcription factors or other regulatory components during transcription. They are involved in the regulation of plant growth and development, and responses to various stresses [29]. In order to understand the putative roles of *Gh2ODD* genes, we selected the 2 kb region upstream of the gene start codons and used PlantCARE software to predict the CREs in *Gh2ODDs*. The promoter regions of *Gh2ODDs* contained light-responsive elements, hormone-responsive elements, growth and stress-related *cis*-acting elements, and a variety of elements with unknown function (Fig. 6). More than half of the Gh2ODD members contained CREs of MYB, MYC, Box4, ARE, ERE, G-Box, GT1-motif, STRE, ABRE, as-1 motif, CGTCA-motif, TGACG-motif, TCT-motif, LTR, W box, WUN-motif and TCA (Table S4). These data highlight the possible important roles of *Gh2ODD* genes in diverse biological processes in cotton.

When plants are under stressful condition, the expression of many genes related to the stress will be induced, and some physiology and biochemistry properties will change in plants. Fourteen CREs involved in stress response were found to be abundant in the promoters of *Gh2ODDs*. They included low temperature response elements, anaerobic inducible response elements, defense and stress response elements, drought inducible response elements and wound response elements (Table S4). Of these, MYB (drought response) and MYC (low temperature response) elements were enriched, accounting for about 96% of the total elements (Table S4). Anaerobic induction response elements and stress response elements accounted for 86 and 73% of Gh2ODD members, respectively. In addition, there were 13 elements involved in hormone response. They are response elements for ethylene, abscisic acid, methyl jasmonate, salicylic acid, gibberellin and auxin. Ethylene- and abscisic acid-responsive elements were widely distributed in Gh2ODD members, accounting for 81 and 71% of the total of elements, respectively, indicating that Gh2ODD may be essential for the signal transduction of ethylene and abscisic acid (Table S4). The *Gh2ODDs* also contained meristem expression (CCGTCC-box), endosperm expression (GCN4_motif), and circadian regulated response elements (circadian). Additionally, there were a large number of elements with unknown functions or specific functions in the *Gh2ODDs*. AAGAA-motif element, box S element, and CARE element were *cis*-elements with unknown functions. About 68% of Gh2ODD family members contained AAGAA-motif elements, pointing to the possible important roles in *Gh2ODDs*. However their functions had not yet been reported. Besides, 38% of the promoters of *Gh2ODDs* contained a specific element 02-site, which may be related to the regulation of zein metabolism (Table S4).

**Fig. 7:**
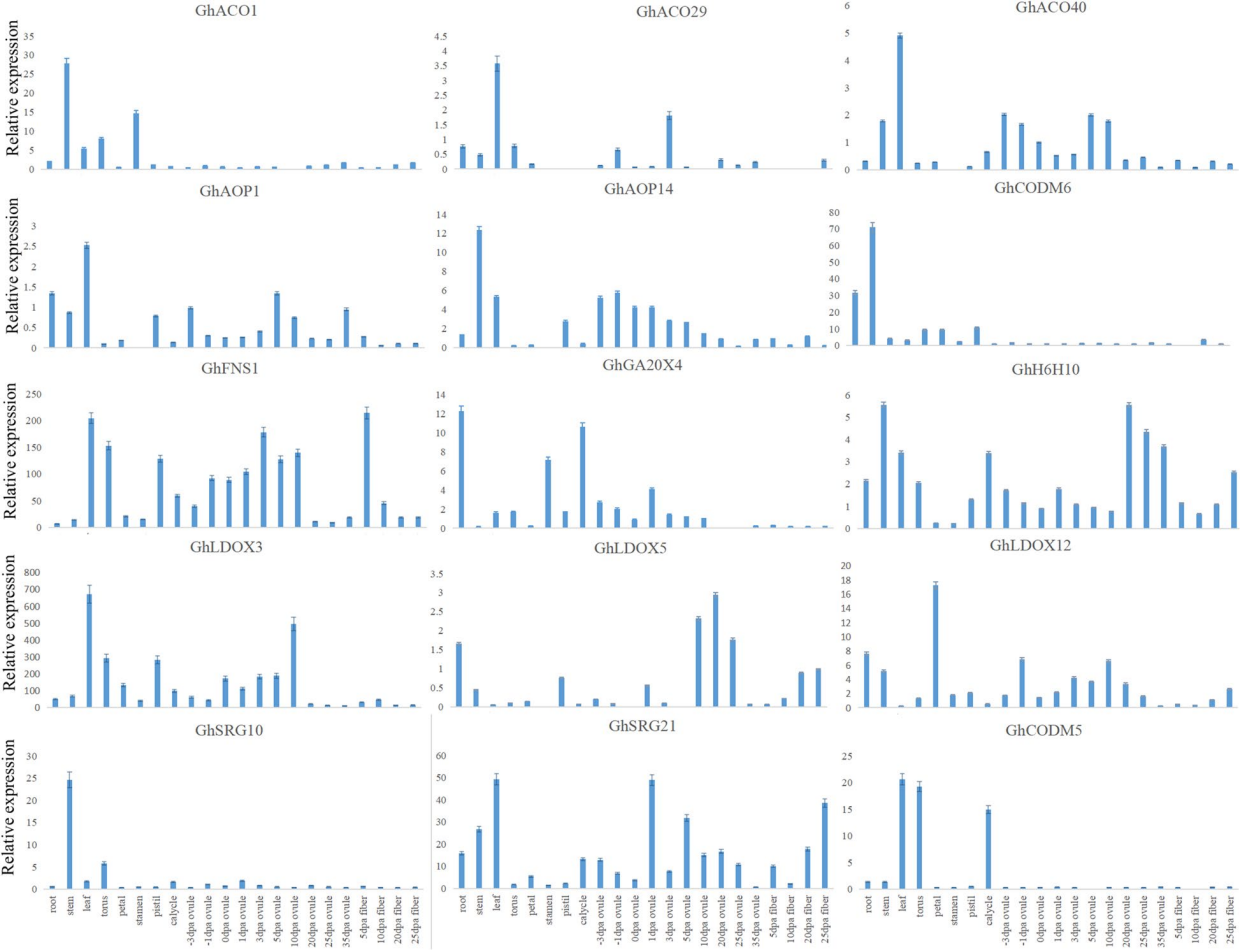
Tissue specific expression profilings of different *Gh2ODDs* in the stem, root, stamen, leaf, pistil, petal, ovule and fiber. Error bars show standard deviations among three independent biological replications

We analyzed the expression levels of *Gh2ODDs* in 22 different tissues, and found that different Gh2ODD members had diverse expression patterns during cotton development. Most *Gh2ODDs* were expressed in various tissues. Yet, *GhP4H10*, *GhP4H12* and *GhP4H1* in the GhP4H subfamily were not expressed in the torus, and *GhP4H10* and *GhP4H1* were not expressed in the stem. GhACO subfamily member *GhACO1* were highly expressed in the stem, and *GhACO49*, *GhACO26*, *GhACO50* and *GhACO35* were strongly expressed in the calycle. The expression levels of GhF6H subfamily members in each tissue were lower than those of other subfamily members. The transcriptional levels of *GhH6H7*, *GhCODM6*, *GhSRG19*, *GhSRG10*, and *GhACO34* in stems were dozens of times higher than those in other tissues. The transcript abundances of *GhFNS1*, *GhLDOX3*, *GhCODM5*, *GhSRG21*, *GhACO40*, *GhACO53*, and *GhACO29* in leaves were several times or even dozens of times of those in other tissues. The expression levels of GhAOP subfamily genes were relatively low in various tissues, and only few members were highly expressed in specific tissues, For instance *GhAOP40*, *GhAOP12*, *GhAOP38*, *GhAOP4*, *GhAOP49*, *GhAOP24*, *GhAOP17*, *GhAOP19*, *GhAOP20*, *GhAOP21*, *GhAOP22*, *GhAOP43* and *GhAOP43* had high expression levels in stems. The mRNA abundances of *GhAOP10*, *GhAOP36*, *GhAOP1* and *GhAOP30* were enriched in leaves. The transcriptional levels of *GhDAO2*, *GhAOP13*, *GhAOP41* and *GhAOP23* were high in petals. *GhGA20OX49* and *GhGA20OX22* showed significantly higher expression levels in petals and stamens than in other tissues (Figs. 6 and 7).

**Fig. 8:**
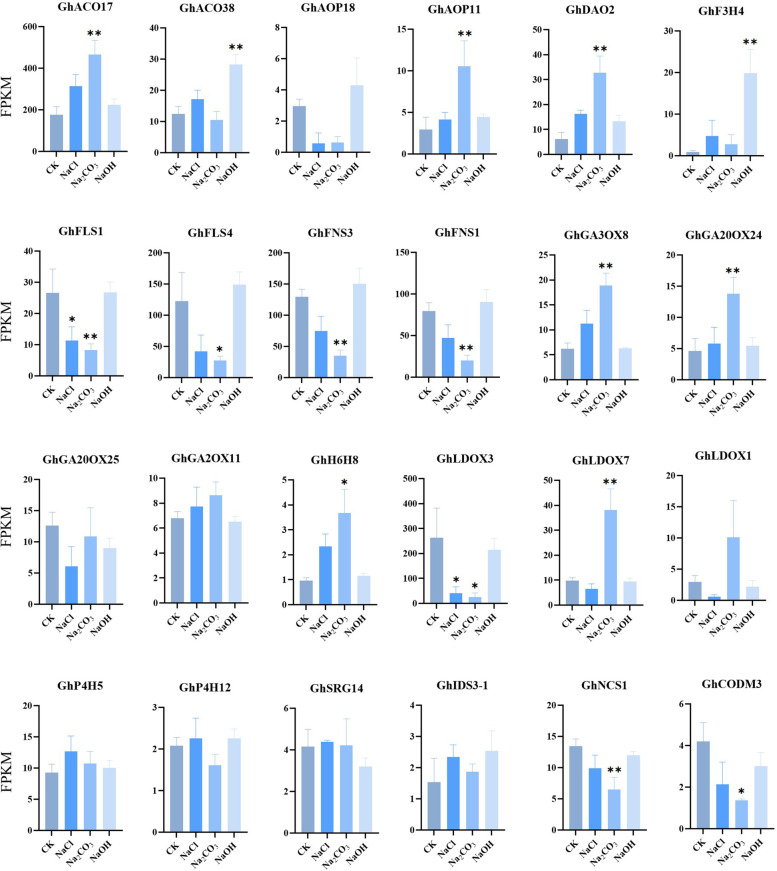
Expression patterns of *Gh2ODD* genes under different saline-alkali stress (100 mM NaCl, 50 mM Na_2_CO_3_and 0.125 mM NaOH). Statistical analyses were performed by Student’s *t*-test (*P < 0.05 and **P < 0.01)

**Fig. 9:**
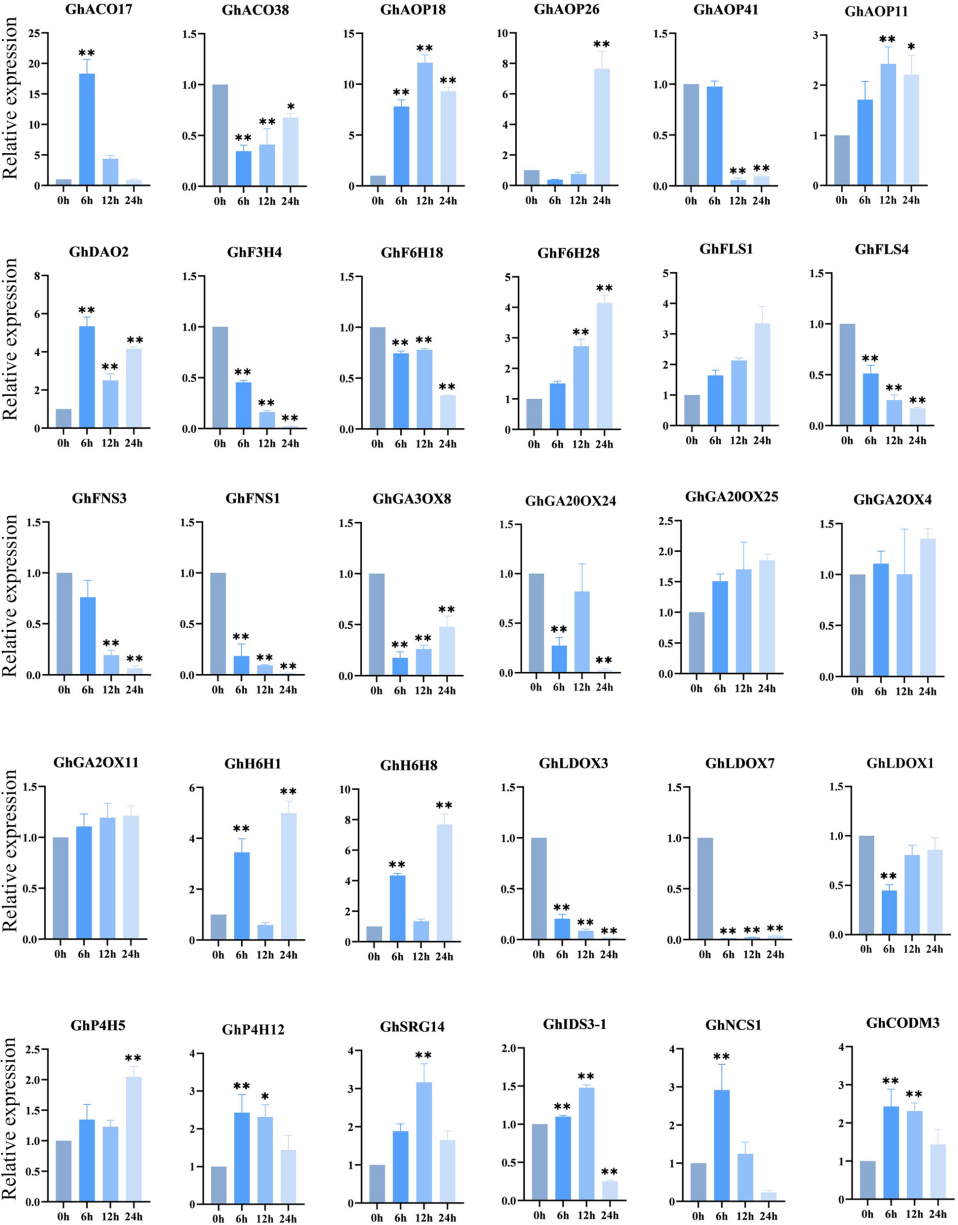
Expression analysis of *Gh2ODDs* in response to alkaline stress in leaves by qRT-PCR. Cotton seedlings were treated with Na_2_CO_3_ stress. The mean values were from three independent biological replicates. Statistical analyses were performed by Student’s *t*-test (*P < 0.05 and **P < 0.01)

We also investigated the gene expression patterns of Gh2ODD genes in cotton response to cold, heat, salt, and PEG stress. Some of the family members showed significant changing trends in response to one of the stresses. *GhP4H2*, *GhP4H4*, *GhP4H7*, *GhP4H14*, *GhP4H15, GhACO26, GhACO49, GhF6H3, GhF6H12, GhSRG14* and *GhSRG21* were significantly down regulated under cold stress and only responded to cold stress. *GhF3H1, GhF3H14, GhACO3, GhACO9* and *GhF6H2* could respond to two stresses. *GhP4H8*, *GhP4H9*, *GhP4H11, GhACO12, GhACO14, GhACO21, GhACO32, GhACO35*, *GhFNS4, GhIDS3-1, GhIDS3-3* and *GhNCS1* responded to three stresses. *GhFNS1, GhFNS2, GhFNS3, GhF3H4, GhF3H11, GhLDOX3, GhLDOX9, GhFLS1, GhFLS3, GhFLS4, GhCODM1, GhACO10, GhACO13, GhACO17, GhACO28, GhACO37*, *GhACO39*, *GhACO28* and *GhAOP43* responded to two stresses. Members of the same subfamily had different response patterns to four abiotic stresses. The results showed that although genes in the same subfamily had similar motifs, they were functionally different. (Fig. 6, Table S5). Transcriptome data also showed that most Gh2ODD family members had different degrees of response under different abiotic stresses (Fig. 8). Most of the *Gh2ODD* genes had significant up-regulated expression or up-regulated expression trend under Na_2_CO_3_ stress, indicating that Gh2ODD family play important roles in cotton response to Na_2_CO_3_ stress. In order to determine whether Gh2ODD indeed have response to Na_2_CO_3_ stress, we measured the expression of multiple *Gh2ODD*s in cotton variety Zhong 9807 after treatment with Na_2_CO_3_ for 0, 6, 12 and 24 h by qRT-PCR. A majority of members in the same subfamily had different expression patterns under alkaline stress (Fig. 9). For example, GhACO subfamily member *GhACO17* was up-regulated while *GhACO38* was down-regulated after alkali stress. Some members in the subfamilies of GhAOP, GhF6H, GhFLS and GhFNS also displayed similar expression patterns to those in GhACO. The transcription levels of *GhLDOX1*, *GhLDOX3* and *GhLDOX7* reduced upon alkali treatment. By contrast, those of *GhSRG14*, *GhCODM3* markedly increased under alkali stress. Therefore, most *Gh2ODDs* may function in cotton responding to alkali stress.

### Interaction network of 2ODD proteins

**Fig. 10:**
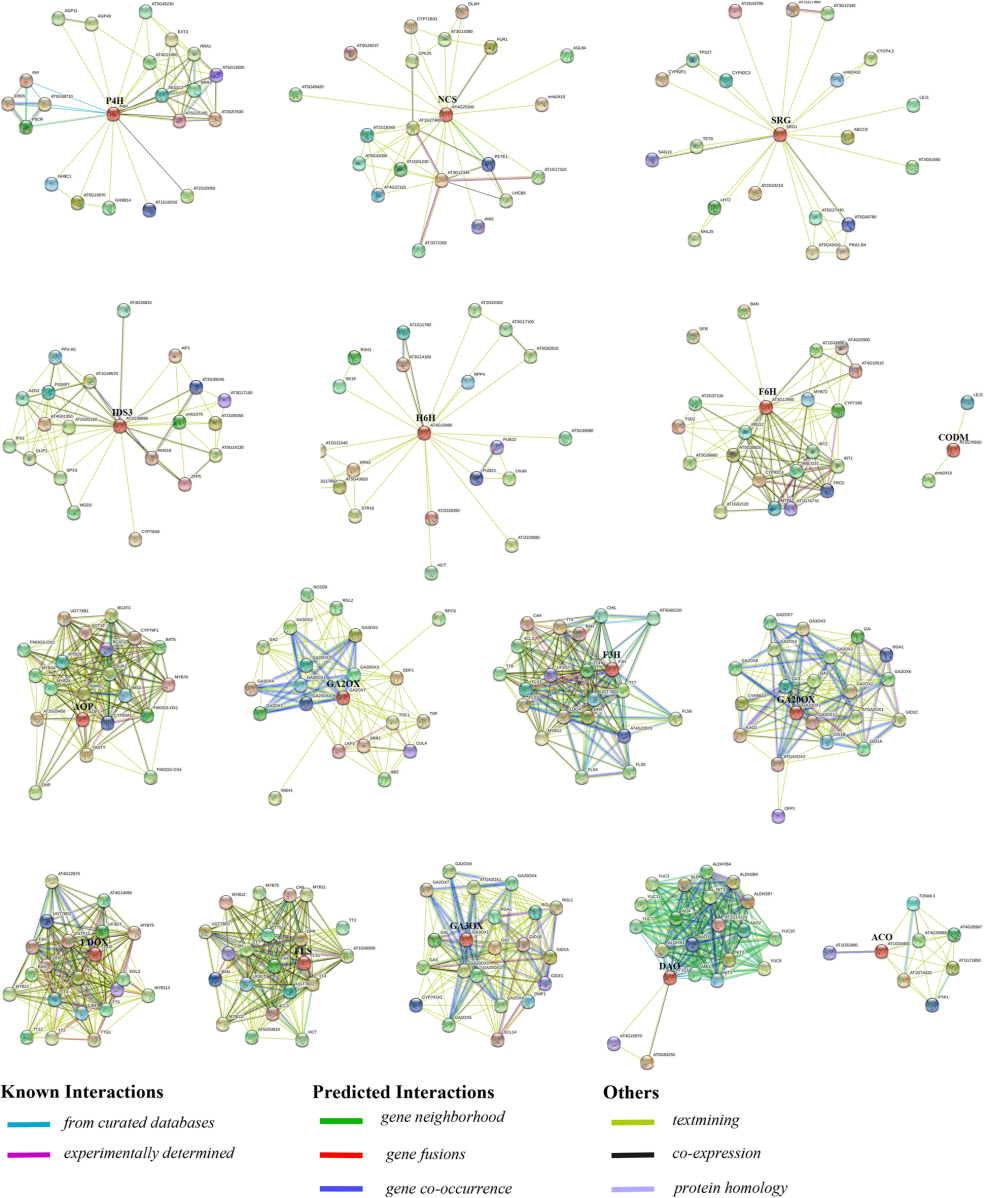
Protein–protein interaction analysis of 2ODD proteins. Protein–protein interaction network produced by the STRINGV9.1. Each node represents a protein and each edge represents an interaction. They are colored by evidence type

Protein interaction network analysis of the orthologs of Gh2ODDs in *Arabidopsis* was performed using the STRING software (Fig. 10). The interactions of P4H protein were predicted with AGP11, AGP40, RRA1, RRA3, P5CR, PIP and ERD5 protein. NCS protein interacted with CPK25, CYP71B31, PETE1, DLAH and FUR1 protein. SRG protein interacted with CYP82C3, CYP82F1, CYP4;1 and ABCC9 protein. IDS3 protein interacted with AIF1, RHS19, SPX3, MGD2 and CYP78A9 protein. H6H protein interacted with RSH1, SK19, RPP4 and XRN3 protein. CODM proteins interacted with LEJI protein. F6H protein interacted with BAN, DFR, TSD2, CYP82C4, CYP71B5, ABCG37 and IRT2 protein. AOP protein interacted with CYP83A1, TASTY, MAM1, SUR1 and IMS2 protein. DAO protein interacted with AAO1, AAO2, AMI1, NIT1, YUC7 and YUC11 protein. ACO protein interacted with PTP1 protein. There was a pairwise interaction among the members in LDOX, FLS, F3H subfamily. The GAOX subfamily proteins GA2OX, GA20OX, and GA3OX interacted reciprocally in pairs. This suggests that 2ODD proteins likely interact mutually to regulate various stress responses. The members in other subfamilies interacted with the proteins other than 2ODDs. P4H proteins interacted with multiple glycosyl hydrolase proteins (AT5G15870, AT1G18310, GH9C1 and GH9B14).

### Cotton plants with silenced *GhLDOX3* by VIGS were insensitive to Na_2_CO_3_ stress

**Fig. 11:**
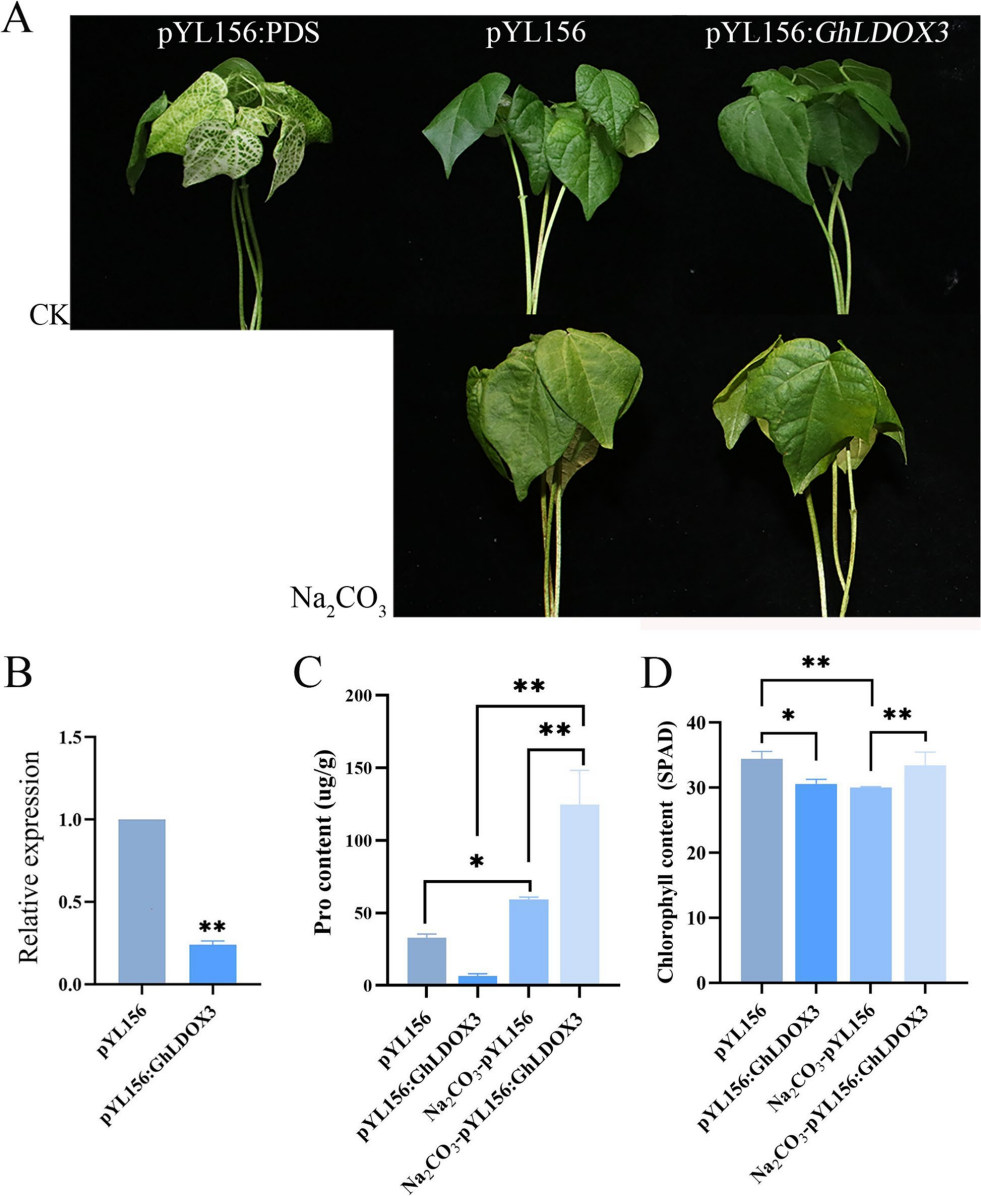
Silencing *GhLDOX3* via VIGS reduced sensitivity to Na_2_CO_3_ stress. **A** The phenotype of cotton after *GhLDOX3* gene silencing under Na_2_CO_3_ stress. pYL156:PDS as a positive control, pYL156 was a negative control (containing empty vector), and pYL156:GhLDOX3 shows a VIGS plant silencing *GhLDOX3*. **B** The expression levels of *GhLDOX3* by qRT-PCR under Na_2_CO_3_ stress. **C** Proline (Pro) contents. **D** Chlorophyll contents. VIGS plants containing vectors pYL156 and pYL156:GhLDOX3 were treated with 0 and 50 mM Na_2_CO_3_ for 24 h. Then, the parameters above were assayed in leaves. Statistical analyses were performed by Student’s *t*-test (*P < 0.05 and **P < 0.01)

To examine whether *GhLDOXs* play roles in responding to Na_2_CO_3_ stress, we studied the functions of a low-expressed gene *GhLDOX3* from transcriptome data by virus-induced gene silencing (VIGS). The phytoene desaturase (PDS) gene, which encodes the Mg-chelatase subunit I (CHLI), was firstly silenced by the method of an *Agrobacterium* infiltration-based VIGS. The leaves of the pYL156:PDS VIGS plants exhibited clear chlorosis phenotypes (Fig. 11A), indicating the efficiency of the VIGS system. Then, cotton leaves were infiltrated by *Agrobacterium* containing the pYL156:*GhLDOX3* vector or the empty vector pYL156. After 2 weeks of growth, the expression levels of *GhLDOX3* showed about 70% decrease in pYL156:*GhLDOX3* compare to pYL156 plants (Fig. 11B). Of note, the leaves from pYL156 seedlings were wilted and light green compared with those from pYL156:*GhLDOX3* plants after treatment with 50 mM Na_2_CO_3_. The proline levels in leaves of pYL156:*GhLDOX3* were considerably higher than those of pYL156 plants under Na_2_CO_3_ stress (Fig. 11C). The chlorophyll content of pYL156:*GhLDOX3* was increased significantly compared to pYL156 under Na_2_CO_3_ stress (Fig. 11D). These results suggest that GhLDOX3 plays a negative role in Na_2_CO_3_ stress tolerance in cotton.

## Discussion

2ODD is the second largest oxidase family in plants and is involved in various oxidative reactions, including hydroxylation, demethylation, dehydrogenation, halogenation and demethylation [30]. In the primary metabolism of plants, 2ODD members are involved in the synthesis of plant growth regulators such as gibberellin and ethylene. In secondary metabolism, 2ODDs play roles in the biosynthesis of flavonoids, alkaloids, terpenoids [31–33], etc. Secondary metabolites (phenylpropanoids, quinones, flavonoids, alkaloids, etc.) are gradually produced in plants during the long-term evolution process for enhancing stress resistance and chemical defense functions. As important secondary metabolites of polyphenols, flavonoids are widely distributed among mosses, liverworts, and vascular plants including angiosperms. However, flavonoids have not been found in lower algae and microorganisms [34]. Plants produce excessive flavonoids to cope with biotic and abiotic stresses, such as the attack of pathogenic microorganisms insects, ultraviolet radiation, drought, etc. [8–10]. 2ODD in plants is divided into three categories, called DOXA, DOXB and DOXC. The DOXA class contains the plant homologs of *Escherichia coli* AlkB, which are involved in the alkylation of nucleic acids and the oxidative demethylation of histones [35]. The DOXB class modulates the levels of proline 4-hydroxyl in cell wall protein synthesis. DOXCs affect plant hormone metabolism and biosynthesis of secondary metabolites, such as flavonoids, alkaloids and terpenoids. It is worth mentioning that most of the 2ODDs from land plants are classified as DOXC classes, and their evolution and expansion lead to the generation of multiple specialized metabolites in plant responding to environmental stresses [36].

Active prolyl 4-hydroxylase is a tetramer consisting of two α subunits and two β subunits, with α subunits playing a major role. The α subunit binds to Fe^2+^, α-ketoglutarate and ascorbic acid, and has a hydroxylation active site. The β subunit is also an important component of prolyl 4-hydroxylase and has protein disulfide isomerase activity [37]. Members of the GhACO subfamily possess the activity of 1-aminocyclopropane-1-carboxylic acid oxidase (ACO), which is involved in the biosynthesis of ethylene [38]. The GhF6H subfamily has the function of feruloyl COA 6-hydroxylase1 (F6H), which is the control enzyme of scopolamine biosynthesis [39]. The GhH6H subfamily members have the function of hyoscyamine 6β-hydroxylase, which catalyzes the formation of scopolamine from hyoscyamine via two consecutive steps [40]. GhIDS3 subfamily members are involved in the biosynthesis of phytosiderophore in mugineic acid family, which encodes a dioxygenase that catalyzes the hydroxylation step from 2'-deoxymugineic acid (DMA) to mugineic acid (MA) [41]. GhNCS has norcoclaurine synthase (NCS) activity, and is involved in the synthesis of benzylisoquinoline alkaloid (BIA) [42]. GhSRG subfamily is a new member of 2ODD, having some aging-related genes [43]. Codeine demethylases (CODMs) of the GhCODM subfamily are responsible for the last two steps of codeine biosynthesis, converting codeine to morphine [44]. The GhAOP subfamily possesses AOP enzymatic activity and catalyzes the conversion of methylsulfinylalkyl glucosinolates to alkenyl glucosinolates [45]. GhGAOX subfamily members are key oxidases in gibberellin biosynthesis. GhLDOX subfamily is a key enzyme at the end of the anthocyanin synthesis pathway. It can convert colorless-anthocyanin into unmodified colored anthocyanin [46]. GhFLS subfamily catalyzes the formation of flavonols from dihydroflavonols [47]. GhF3H subfamily catalyzes the 3-beta-hydroxylation of 2S-flavanones to 2R, 3R-dihydroflavonols which are intermediates in the biosynthesis of flavonols, anthocyanidins, catechins and proanthocyanidins in plants [48]. Flavonoid synthase in GhFNS subfamily member is the key enzyme in the conversion of flavanones to flavones [49] The GhDAO subfamily is essential for auxin catabolism and maintenance of auxin homeostasis in reproductive organs [50].

A gene family is a group of genes consisting of two or more copies of the same ancestral gene produced by gene duplication. Gene duplication is a major source of formation of new genes and gene functions and plays a key role in trait diversity and speciation [51]. Genome changes generally occur during the formation of polyploid plants, and the changes include duplication between genes, rearrangement of chromosomes and changes in the number of genes. The evolution of gene families mainly includes segmental duplication, tandem duplication and whole genome duplication. Among them, whole-genome duplication plays an important role in gene amplification [52–54].

In this study, 1124 *2ODD* genes were identified in four species (*G. hirsutum*, *G. arboreum*, *G. raimondii*, *G. barbadense*). Of these, 336 were found in *G. hirsutum*. However, only 130 and 114 2ODDs were detected from *Arabidopsis* and *Oryza*, respectively [36]. Overall, *Gh2ODD* genes were conservative during evolution, but, the events of gene loss and unbalanced evolution between genes occurred. The possible reason is the doubling of the entire genome when two diploids (*G. arboreum* and *G. raimondii*) are hybridized to form an allotetraploid (*G. hirsutum* and *G. barbadense*). An increase in the number of genes contributes gene evolution. Whole-genome duplication, tandem duplication and segmental duplication also play an important role in family expansion [55]. The existence of gene duplication can promote new genes to acquire new functions, and tandem duplication can promote the changes in gene structure and function more quickly [56]. The combined effect leads to functional progress in genetic diversity and environmental adaptability.

The *Ka/Ks* ratio can reflect whether there is selective pressure acting on the gene encoding a certain protein. In the study of gene family evolution, synonymous substitutions refer to nucleotide variations that do not lead to amino acid changes, so synonymous substitutions are generally not affected by natural selection and are selected as neutrals. Non-synonymous substitutions refer to those nucleotide variations that result in coding amino acid variations. In general, non-synonymous substitution causes amino acid changes that may alter protein conformation and function. Thus, adaptive changes may occur, which may be favorable or unfavorable (usually unfavorable) [57]. Synonymous substitution does not change the composition of the protein, and plant genes are therefore not subject to natural selection (here we ignore the effect of codon preference). It is generally considered that when *Ka* > *Ks* or *Ka/Ks* > 1, the homologous gene is positively selected. *Ka* = Ks or *Ka/Ks* = 1, the homologous gene is neutrally selected. *Ka* < *Ks* or *Ka/Ks* < 1, homologous genes are affected by purification selection.

From the functional point of view, the new gene formed by replication is not seriously differentiated from the source gene, and is relatively conservative in evolution, having relatively stable structure and consistent function (Table S3). 57 pairs of homologous genes with a *Ka/Ks* ratio greater than 1, it is speculated that the duplicated gene pairs of these two tetraploid genomes may have evolved to acquire new and different functions to adapt to the environment. Since most of the *Ka/Ks* values are less than 1 (Fig. 4), it can be speculated that the cotton 2ODD gene family has undergone a strong purifying selection pressure with limited functional differentiation after segmental duplication and WGD.

Gene DNA is divided into coding region and non-coding region, and the coding region contains exons and introns. In plants, most genes are interrupted by single or multiple exons/introns, and their arrangement and location can be used to study the evolutionary relationships among gene family members. Generally, genes of the same subclass have similar exon/intron arrangements in terms of intron number and exon length. Studies have shown that exons/introns are related to their related biological functions [58].

In previous studies, genes with few or no introns were thought to have higher expression levels in plants [59, 60]. To respond to various stresses in a timely manner, genes must be activated rapidly, and compact gene structures with fewer introns can aid in stress-responsive gene activation.

LDOX plays a key regulatory role in a variety of plant biological processes. The leaves of the LDOX allele mutant *tds4* lack anthocyanins in *Arabidopsis*. Transmission electron microscopy revealed that *tds4* endothelial cells had multiple small vacuoles, instead of a large central vacuole as observed in the WT [61]. Under different abiotic stresses, *Gh2ODD* genes showed different expression trends. Different *Gh2ODD* genes also have diverse expression patterns during development, indicating that *Gh2ODD* genes may be involved in these processes through different mechanisms. *Gh2ODD*s play more important roles in cotton responding to salt and PEG stress than to cold and heat stress. The higher concentration of Na^+^ in saline-alkali soil solutions leads to hyperosmotic stress, which hinders plants from absorbing water and nutrients from the soil. Drought stress affects nutrient availability and transport [62, 63]. The expressions of 6 members of the *GhP4H* subfamily were down-regulated under cold stress, and 5 *GhP4Hs* were up-regulated under PEG stress. Among these *GhP4Hs*, *GhP4H9* and *GhP4H21* were down-regulated under cold stress and up-regulated under PEG stress. It was found that *GhACO32* was significantly down-regulated under salt stresses. This is consistent with the expression trend of *TaACO1* under salt stress [64]. In the GhF6H subfamily, only *GhF6H2*, *GhF6H3*, *GhF6H16* and *GhF6H17* serve roles in responding to stress. The expression levels of each member in tissues are very low. It may be that members of the GhF6H subfamily play a negative regulatory role in development or do not modulate plant growth and developmental in plants. In the GhFNS subfamily, *GhFNS4*, *GhFNS1*, *GhFNS2* and *GhFNS3* were significantly down-regulated under the four stress treatments above. In the GhLDOX subfamily, the expression of *GhLDOX3* was up regulated at 1 h and significantly decreased at 3, 6 and 12 h under cold, salt and PEG stress, but continued to decrease under heat stress, indicating that *GhLDOX3* has a different response mechanism to stress. Other *GhLDOXs* did not change significantly in expression under stress treatment. Collectively, these results imply that *GhLDOX3* may play an important role in cotton response to saline-alkali stress. Most members of the GhGAOX subfamily do not change in expession upon stress, only *GhGA2OX11* and *GhGA2OX7* were up-regulated under heat and cold stress, respectively. They had high expression levels in calycle and stamens. Together, these data suggest that many 2ODDs in plants may regulate different cellular processes, and they likely interact, restrict and coordinate with each other, and jointly modulate plant growth, development and stress response.

As a major abiotic stress limiting agricultural production, alkali stress severely disrupts plant physiology, biochemistry, metabolism, and development, and inhibits plant growth [65]. Studies show that alkali stresses significantly inhibit cotton growth. The reason may be that a high concentration of Na^+^ leads to osmotic imbalance, membrane dysfunction, increased production of ROS, as well as the disruption of plant nutrient metabolism by high pH under alkali stress, and thus affecting cell division and growth [66, 67]. Soil salinization and alkalization frequently co-occur, but compared with salt stress, alkali stress causes greater damage to plants [3]. In this study, it was found that the expression patterns of genes were different under salt and alkali stress, indicating that the mechanism of action of alkali stress and salt stress is different (Fig. 9). Presumably, alkali stress with the high pH will severely disturb cell pH stability, destroy cell membrane integrity, and decrease root vitality and photosynthetic function [65].

Pro is a ubiquitous protective substance in plants. It is a low molecular weight cyclic amino acid and is known to provide osmotic adjustments in plants under stressful environments. Plants adjust the content of Pro under stress to change the osmotic pressure, maintain the stability and integrity of the cell membrane, ensure the normal physiological activity of enzymes, and maintain the intake and maintenance of water [68]. Chlorophyll is a typical representative indicator of photosynthesis. As the important photosynthetic pigment, it can reflect the photosynthetic energy efficiency [69]. After Na_2_CO_3_ stress, the Pro and chlorophyll contents of pYL156:*GhLDOX3* were evidently more than pYL156 (Fig. 11C and D). Combined with the phenotype of Fig. 11A, we speculated that the *GhLDOX3* is involved in the response to Na_2_CO_3_ stress in cotton.

## Conclusion

There were 336 Gh2ODDs in *G. hirsutum*. They were divided into 15 subfamilies. *Ka / Ks* and collinearity analyses indicated that Gh2ODDs experienced strong purifying selection pressure, and segment duplication and whole-genome duplication played important roles in gene expansion. Tetraploid *G. hirsutum* is derived from the two diploid *Gossypium* species (Ga & Gr), during the evolution process, gene loss and chromosome fusion events occurred. Silencing *GhLDOX3* led to more Pro and chlorophyll in cotton, suggesting that *GhLDOX3* is involved in the response to Na_2_CO_3_ stress in cotton.

## Material and methods

### Data acquisition

Genome files of four cotton species, *Gossypium arboreum* (CRI), *Gossypium raimondii* (JGI), *Gossypium barbadense* (ZJU) and *Gossypium hirsutum* (NAU) were obtained from Cotton Functional Genomic Database (CottonFGD) (https://cottonfgd.org/) [70].

### Identification of 2ODD family members

To identify the members of the 2ODD gene family, the protein sequences and genome annotations of 2ODDs from two tetraploid (*G. barbadense* & *G. hirsutum*) and two diploid (*G. arboreum* & *G. raimondii*) species were downloaded from Cotton Functional Genomic Database (CottonFGD) (https://cottonfgd.org/) [70]. The HMMER 3.0 software was used with default settings and parameters to obtain the sequences of 2ODDs containing Pfam PF14226 and PF03171 domains. We removed the redundant genes with an e-value greater than 1E-05. We further examined various biophysical properties of 2ODD*s* including protein and genomic lengths, molecular weights (MWs), isoelectric points (pIs), the number of exons and other biophysical properties from CottonFGD (https://cottonfgd.org/).

### Phylogenetic analysis and sequences alignments

The full-length amino acid sequences of 2ODDs from *G. hirsutum*, *G. arboreum*, *G. raimondii* and *G. barbadense *were downloaded, and multiple sequence alignment analysis was performed using MEGA software (version 7.0) and ClustalW program. Subsequently, we constructed the phylogenetic tree using neighbor joining (NJ) method and Maximum likelihood method (ML) with 1000 bootstrap replicates, respectively, in MEGA software (version 7.0) [71].

### Gene duplication relationship, selection pressure and collinearity analysis of 2ODD genes

The 2ODDs homologous gene pairs of four cotton species were identified by searching the gene duplication across the four species through the NCBI local blast toolkit combined with the TBtools software under strict criteria (1) alignment length coverage was set over 70%, (2) similarity index in the aligned region was considered at least 80% and (3) for strongly connected genes a minimum of two duplication events were given consideration [72]. Syntenic relationship and collinearity among orthologs/paralogs were examined with the MCScanX software [73]. Duplicated gene pairs belonging to the same genome/subgenome and locating at the same chromosome with a maximum of 200 kb distance between each other are considered as tandem duplication. Selection pressure experienced by each duplicated pair during evolution was calculated according to the rate of non-synonymous (*Ka*) to synonymous (*Ks*) substitution using the *Ka/Ks* calculator 2.0 software [74]. While separation time for duplicated pairs was measured using the following formula, that is *Ks* / 2r, r = 1.5 × 10^–8^, and the unit of separation time was Mya, where M = 10^6^.

### Organization of 2ODD genes on chromosomes of four *Gossypium* species

Physical positions of 2ODD genes in chromosomes from four *Gossypium* species were drawn by the TBtools software [75]. Genomic sequences, coding sequences, Generic Feature Format (gff) files of all of the four species were downloaded from CottonFGD (http://www.cottonfgd.org/) [70]. The positions of *2ODDs* were determined based on the gff files and gene IDs.

### Analysis of the conserved protein motifs and gene structure

Online webtool Multiple Em for Motif Elicitation (MEME) (http://meme-suite.org/) was used to identify the conserved protein motifs [76]. The Gene Structure Display Server program (http://gsds.cbi.pku.edu.cn/) was applied to illustrate the gene structures by aligning the coding sequences with the DNA sequences of *2ODD* genes [77]*.* The phylogenetic tree, gene structure and conserved motifs of *2ODD* genes were visualized and integrated into graphics by TBtools software.

### Analysis of cis elements in promoter regions and gene enrichment and protein interaction network

DNA sequences of 2000 bp in upstream regions of *2ODD* genes were obtained from CottonFGD database (http://www.cottonfgd.org/) as promoters. The PlantCARE (http://bioinformatics.psb.ugent.be/webtools/plantcare/html/) was used for the prediction of *cis*-acting elements in promoter regions of *2ODD* genes. The 2ODD protein interaction network was examined using the STRING online server (https://cn.string-db.org/).

### Tissue-specific expression profilings, stress treatments and qRT-PCR

RNA-Seq data (PRJNA490626) were downloaded from the website (http://grand.cricaas.com.cn/page/tools/expressionVisualization) to examine the relative expression patterns of *2ODDs* under abiotic stress (cold, heat, salt and PEG) with various time periods (0, 1, 3, 6 and 12 for each treatment) and different tissues including the root, shoot, petal, leaf, pistil, stamen, torus and calycle, along with the ovule and fiber [78]. Zhang's transcriptome data under different saline-alkali stress (100 mM NaCl, 50 mM Na_2_CO_3_, 0.125 mM NaOH) were used to determine the expression levels of Gh2ODD family members [79]. The heat map was generated through the TBtools software with FPKM values for relative expression analysis.

*G. hirsutum* accession Zhong 9807 was used in this study. Cotton seedlings at three true leaf stage under normal field conditions were treated by various abiotic stresses and sampled for tissue specific expression analysis. Tissue specific expression profilings of different *Gh2ODDs* in the root, stem, leaf, petal, stamen, pistil, ovule and fiber were assayed. The expression of different *Gh2ODDs* in cotton response to salt (400 mM NaCl), drought (20% PEG), cold (4℃) and heat (37℃) for different time points (1, 3, 6 and 12 h) were analyzed. We used the FastPure® Plant Total RNA lsolation Kit (Nanjing Vazyme Biotech Co., Ltd.) to extract total RNA from all samples of cotton plants which were further used to synthesize cDNA with Prime-Script®RT reagent kit (Takara, China) following manual instruction. All the samples were collected and transferred to liquid nitrogen immediately, saved at – 80℃ for future use.

### Virus‑induced gene silencing (VIGS) experiment

The purified GhLDOX3 fragment was inserted into the empty pYL156 to form the pYL156:*GhLDOX3* vector, with the sites of BamHI and SmaI. The GV3101 strains carrying pYL156 (empty vector), pYL156:*GhLDOX3* (VIGS), pYL156:PDS (positive control), and pYL192 (helper vector) were cultured to OD600 = 1.2. Each mixture was injected into the cotyledons of cotton variety Zhong 9807 plants. After injection, the plants were placed in the dark overnight, and a 16 h light/8 h dark cycle was performed at 25 °C. The plants injected with pYL156 and pYL156:*GhLDOX3* were treated with alkaline treatment after the cotton plants with pYL156:PDS developed an albino phenotype.

## Supplementary Information

**Additional file 1:** **Table S1.** Gene ID and gene renamed in the four cotton species. **Table S2.** Biochemical characteristics of Gh2ODD family. **Table S3.** Pairwise comparison of synonymous and non-synonymous substitutions and tentative divergence times of orthologous 2ODDs among four Gossypium species. **Table S4.** Analysis of cis-acting elements in the Gh2ODD family. **Table S5.** FPKM of members in Gh2ODD who respond to different abiotic stresses.

## Data Availability

The source data underlying the graphs in the main figures are available in Supplementary Tables. Genome files of four cotton species were obtained from Cotton Functional Genomic Database (CottonFGD) (CottonFGD: HomePage).

## Abbreviations

2ODD: 2-Oxoglutarate-dependent dioxygenase
F3H: Flavanone-3-hydroxylase
DFR: Dihydroflavonol-4-reductase
FLS: Flavonol synthase
LDOX: Leucocyanidin dioxygenase
NJ: Neighbor-Joining
ML: Maximum Likelihood

## References

[1] Li J, Pu L, Han M, Zhu M, Zhang R, Xiang Y (2014). Soil salinization research in China: advances and prospects. J Geog Sci.

[2] Wang H, Takano T, Liu S. Screening and evaluation of saline–alkaline tolerant germplasm of rice (*Oryza sativa* L.) in soda saline–alkali soil. Agronomy. 2018;8(10):205. 10.3390/agronomy8100205.

[3] Wang X, Ren H, Wei Z, Wang Y, Ren W (2017). Effects of neutral salt and alkali on ion distributions in the roots, shoots, and leaves of two alfalfa cultivars with differing degrees of salt tolerance. J Integr Agric.

[4] Ashraf J, Zuo D, Wang Q, Malik W, Zhang Y, Abid MA, Cheng H, Yang Q, Song G (2018). Recent insights into cotton functional genomics: progress and future perspectives. Plant Biotechnol J.

[5] Farrow SC, Facchini PJ (2014). Functional diversity of 2-oxoglutarate/Fe (II)-dependent dioxygenases in plant metabolism. Front Plant Sci.

[6] Jaakola L (2013). New insights into the regulation of anthocyanin biosynthesis in fruits. Trends Plant Sci.

[7] Kong J, Chia L, Goh N, Chia T, Brouillard R (2003). Analysis and biological activities of anthocyanins. Phytochemistry.

[8] Ma Y, Ma X, Gao X, Wu W, Zhou B. Light induced regulation pathway of anthocyanin biosynthesis in plants. Int J Mol Sci. 2021;22(20). 10.3390/ijms222011116. 10.3390/ijms222011116 PMC8538450 34681776

[9] Takshak S, Agrawal S. Defence strategies adopted by the medicinal plant *Coleus forskohlii* against supplemental ultraviolet-B radiation: augmentation of secondary metabolites and antioxidants. Plant Physiol Biochem. 2015;97:124–38. 10.1016/j.plaphy.2015.09.018. 10.1016/j.plaphy.2015.09.018 26461242

[10] Crifò T, Puglisi I, Petrone G, Recupero GR, Piero ARL (2011). Expression analysis in response to low temperature stress in blood oranges: implication of the flavonoid biosynthetic pathway. Gene.

[11] Liu H, Liu Z, Wu Y, Zheng L, Zhang G (2021). Regulatory mechanisms of anthocyanin biosynthesis in apple and pear. Int J Mol Sci.

[12] Lila MA, Burton-Freeman B, Grace M, Kalt W (2016). Unraveling anthocyanin bioavailability for human health. Annu Rev Food Sci Technol.

[13] Khoo HE, Azlan A, Tang ST, Lim SM (2017). Anthocyanidins and anthocyanins: colored pigments as food, pharmaceutical ingredients, and the potential health benefits. Food Nutr Res.

[14] Huang H, Hu K, Han K, Xiang Q, Dai S. Flower colour modification of chrysanthemum by suppression of *F3 ' H* and overexpression of the exogenous senecio cruentus *F3 ' 5 ' H* gene. Plos One. 2013;8(11). 10.1371/journal.pone.0074395. 10.1371/journal.pone.0074395 PMC3826725 24250783

[15] He J, Giusti MM (2010). Anthocyanins: natural colorants with health-promoting properties. Annu Rev Food Sci Technol.

[16] Springob K, Nakajima J, Yamazaki M, Saito K (2003). Recent advances in the biosynthesis and accumulation of anthocyanins. Nat Prod Rep.

[17] Wilmouth RC, Turnbull JJ, Welford RW, Clifton IJ, Prescott AG, Schofield CJ (2002). Structure and mechanism of anthocyanidin synthase from *Arabidopsis thaliana*. Structure..

[18] Wang Y, Shi Y, Li K, Yang D, Liu N, Zhang L, Zhao L, Zhang X, Liu Y, Gao L (2021). Roles of the 2-oxoglutarate-dependent dioxygenase superfamily in the flavonoid pathway: a review of the functional diversity of F3H, FNS I, FLS, and LDOX/ANS. Molecules.

[19] Jayaraman K, Sevanthi AM, Sivakumar S, Viswanathan C, Mohapatra T, Mandal PK (2021). Stress-inducible expression of chalcone isomerase2 gene improves accumulation of flavonoids and imparts enhanced abiotic stress tolerance to rice. Environ Exp Bot.

[20] Li C, Liu S, Yao X, Wang J, Wang T, Zhang Z, Zhang P, Chen K (2017). PnF3H, a flavanone 3-hydroxylase from the Antarctic moss *Pohlia nutans*, confers tolerance to salt stress and ABA treatment in transgenic *Arabidopsis*. Plant Growth Regul..

[21] Liang J, He J (2018). Protective role of anthocyanins in plants under low nitrogen stress. Biochem Biophys Res Commun.

[22] Li N, Wang X, Ma B, Wu Z, Zheng L, Qi Z, Wang Y (2021). A leucoanthocyanidin dioxygenase gene (*RtLDOX2*) from the feral forage plant *Reaumuria trigyna* promotes the accumulation of flavonoids and improves tolerance to abiotic stresses. J Plant Res..

[23] Li P, Li YJ, Zhang FJ, Zhang GZ, Jiang XY, Yu HM, Hou BK (2017). The *Arabidopsis* UDP-glycosyltransferases UGT79B2 and UGT79B3, contribute to cold, salt and drought stress tolerance via modulating anthocyanin accumulation. Plant J..

[24] Wang M, Ren T, Huang R, Li Y, Zhang C, Xu Z. Overexpression of an *Apocynum venetum* flavonols synthetase gene confers salinity stress tolerance to transgenic tobacco plants. Plant Physiol Biochem. 2021;162:667–76. 10.1016/j.plaphy.2021.03.034. 10.1016/j.plaphy.2021.03.034 33780740

[25] Jun JH, Xiao X, Rao X, Dixon RA (2018). Proanthocyanidin subunit composition determined by functionally diverged dioxygenases. Nature Plants.

[26] Shi S, Li S, Kang Y, Liu J (2015). Molecular characterization and expression analyses of an anthocyanin synthase gene from *Magnolia sprengeri* pamp. Appl Biochem Biotechnol..

[27] Boss PK, Davies C, Robinson SP (1996). Expression of anthocyanin biosynthesis pathway genes in red and white grapes. Plant Mol Biol.

[28] Paterson AH, Wendel JF, Gundlach H, Guo H, Jenkins J, Jin D, Llewellyn D, Showmaker KC, Shu S, Udall J (2012). Repeated polyploidization of *Gossypium* genomes and the evolution of spinnable cotton fibres. Nature..

[29] Lu Z, Ricci WA, Schmitz RJ, Zhang X (2018). Identification of *cis*-regulatory elements by chromatin structure. Curr Opin Plant Biol..

[30] Loenarz C, Schofield CJ (2008). Expanding chemical biology of 2-oxoglutarate oxygenases. Nat Chem Biol.

[31] Samanani N, Liscombe DK, Facchini PJ (2004). Molecular cloning and characterization of norcoclaurine synthase, an enzyme catalyzing the first committed step in benzylisoquinoline alkaloid biosynthesis. Plant J.

[32] De Luca V, Salim V, Thamm A, Masada SA, Yu F (2014). Making iridoids/secoiridoids and monoterpenoid indole alkaloids: progress on pathway elucidation. Curr Opin Plant Biol.

[33] Vazquez-Flota F, De Carolis E, Alarco A-M, De Luca V. Molecular cloning and characterization of desacetoxyvindoline-4-hydroxylase, a 2-oxoglutarate dependent-dioxygenase involved in the biosynthesis of vindoline in *Catharanthus roseus* (L.) G. Don. Plant Mol Biol. 1997;34(6):935–48. 10.1023/a:1005894001516. 10.1023/a:1005894001516 9290645

[34] Winkel-Shirley B (2001). Flavonoid biosynthesis. A colorful model for genetics, biochemistry, cell biology, and biotechnology. Plant Physiol.

[35] Lam LT, Pickeral OK, Peng AC, Rosenwald A, Hurt EM, Giltnane JM, Averett LM, Zhao H, Davis RE, Sathyamoorthy M (2001). Genomic-scale measurement of mRNA turnover and the mechanisms of action of the anti-cancer drug flavopiridol. Genome Biol.

[36] Kawai Y, Ono E, Mizutani M (2014). Evolution and diversity of the 2–oxoglutarate-dependent dioxygenase superfamily in plants. Plant J.

[37] Friedman L, Higgin JJ, Moulder G, Barstead R, Raines RT, Kimble J. Prolyl 4-hydroxylase is required for viability and morphogenesis in *Caenorhabditis elegans*. Proc Natl Acad Sci USA. 2000;97(9):4736–41. 10.1073/pnas.97.9.4736. 10.1073/pnas.97.9.4736 PMC18302 10781079

[38] Norikoshi R, Niki T, Ichimura K. Differential regulation of two 1-aminocyclopropane-1-carboxylate oxidase (ACO) genes, including the additionally cloned *DcACO2*, during senescence in carnation flowers. Postharvest Biol Technol. 2022;183:111752. 10.1016/j.postharvbio.2021.111752.

[39] Liu S, Zainuddin IM, Vanderschuren H, Doughty J, Beeching JR. RNAi inhibition of feruloyl CoA 6′-hydroxylase reduces scopoletin biosynthesis and post-harvest physiological deterioration in cassava (*Manihot esculenta Crantz*) storage roots. Plant Mol Biol. 2017;94(1):185–95. 10.1007/s11103-017-0602-z. 10.1007/s11103-017-0602-z PMC5437147 28315989

[40] Kluza A, Wojdyla Z, Mrugala B, Kurpiewska K, Porebski PJ, Niedzialkowska E, Minor W, Weiss MS, Borowski T (2020). Regioselectivity of hyoscyamine 6β-hydroxylase-catalysed hydroxylation as revealed by high-resolution structural information and QM/MM calculations. Dalton Trans.

[41] Kobayashi T, Nakanishi H, Takahashi M, Kawasaki S, Nishizawa N-K, Mori S. In vivo evidence that *Ids3* from *Hordeum vulgare* encodes a dioxygenase that converts 2′-deoxymugineic acid to mugineic acid in transgenic rice. Planta. 2001;212(5):864–71. 10.1007/s004250000453. 10.1007/s004250000453 11346963

[42] Luk LY, Bunn S, Liscombe DK, Facchini PJ, Tanner ME (2007). Mechanistic studies on norcoclaurine synthase of benzylisoquinoline alkaloid biosynthesis: an enzymatic Pictet− Spengler reaction. Biochemistry.

[43] Zentgraf U, Jobst J, Kolb D, Rentsch D. Senescence-related gene expression profiles of rosette leaves of *Arabidopsis thaliana*: leaf age versus plant age. Plant Biol. 2004;6(02):178–83. 10.1055/s-2004-815735. 10.1055/s-2004-815735 15045669

[44] Farrow SC, Facchini PJ. Dioxygenases catalyze *O*-demethylation and *O*, *O*-demethylenation with widespread roles in benzylisoquinoline alkaloid metabolism in opium poppy. J Biol Chem. 2013;288(40):28997–9012. 10.1074/jbc.M113.488585. 10.1074/jbc.M113.488585 PMC3789997 23928311

[45] Kliebenstein D. Gene duplication and the diversification of secondary metabolism: side chain modification of glucosinolates in *Arabidopsis thaliana*. Plant Cell. 2001;13:681–93. 10.1105/tpc.109.067611. 10.1105/tpc.13.3.681 PMC135509 11251105

[46] Pelletier MK, Burbulis IE, Winkel-Shirley B. Disruption of specific flavonoid genes enhances the accumulation of flavonoid enzymes and end-products in *Arabidopsis* seedlings. Plant Mol Biol. 1999;40(1):45–54. 10.1023/a:1026414301100. 10.1023/a:1026414301100 10394944

[47] Owens DK, Alerding AB, Crosby KC, Bandara AB, Westwood JH, Winkel BS. Functional analysis of a predicted flavonol synthase gene family in *Arabidopsis*. Plant Physiol. 2008;147(3):1046–61. 10.1104/pp.108.117457. 10.1104/pp.108.117457 PMC2442520 18467451

[48] Xiong S, Tian N, Long J, Chen Y, Qin Y, Feng J, Xiao W, Liu S. Molecular cloning and characterization of a flavanone 3-hydroxylase gene from *Artemisia annua* L. Plant Physiol Biochem. 2016;105:29–36. 10.1016/j.plaphy.2016.04.005. 10.1016/j.plaphy.2016.04.005 27070290

[49] Wu J, Wang X-C, Liu Y, Du H, Shu QY, Su S, Wang LJ, Li SS, Wang LS. Flavone synthases from *Lonicera japonica* and *L. macranthoides* reveal differential flavone accumulation. Sci Rep. 2016;6(1):19245. 10.1038/srep19245. 10.1038/srep19245 PMC4709722 26754912

[50] Zhao Z, Zhang Y, Liu X, Zhang X, Liu S, Yu X, Ren Y, Zheng X, Zhou K, Jiang L (2013). A role for a dioxygenase in auxin metabolism and reproductive development in rice. Dev Cell.

[51] Lynch M, Conery JS (2000). The evolutionary fate and consequences of duplicate genes. Science.

[52] Chen ZJ, Sreedasyam A, Ando A, Song Q, De Santiago LM, Hulse-Kemp AM, Ding M, Ye W, Kirkbride RC, Jenkins J (2020). Genomic diversifications of five *Gossypium* allopolyploid species and their impact on cotton improvement. Nat Genet..

[53] He P, Zhang Y, Xiao G (2020). Origin of a subgenome and genome evolution of allotetraploid cotton species. Mol Plant.

[54] Xu G, Guo C, Shan H, Kong H (2012). Divergence of duplicate genes in exon–intron structure. Proc Natl Acad Sci.

[55] Hanada K, Zou C, Lehti-Shiu M, Shinozaki K, Shiu S (2008). Importance of lineage-specific expansion of plant tandem duplicates in the adaptive response to environmental stimuli. Plant Physiol.

[56] Lehti-Shiu M, Zou C, Hanada K, Shiu S (2009). Evolutionary history and stress regulation of plant receptor-like kinase/pelle genes. Plant Physiol.

[57] Yang Z (1997). PAML: a program package for phylogenetic analysis by maximum likelihood. Comput Appl Biosci.

[58] Malik WA, Wang X, Wang X, Shu N, Cui R, Chen X, Wang D, Lu X, Yin Z, Wang J (2020). Genome-wide expression analysis suggests glutaredoxin genes response to various stresses in cotton. Int J Biol Macromol.

[59] Chung BY, Simons C, Firth AE, Brown CM, Hellens RP (2006). Effect of 5’UTR introns on gene expression in *Arabidopsis thaliana*. BMC Genomics..

[60] Ren X, Vorst O, Fiers M, Stiekema W, Nap J (2006). In plants, highly expressed genes are the least compact. Trends Genet.

[61] Abrahams S, Lee E, Walker AR, Tanner GJ, Larkin PJ, Ashton AR (2003). The *Arabidopsis TDS4* gene encodes leucoanthocyanidin dioxygenase (LDOX) and is essential for proanthocyanidin synthesis and vacuole development. Plant J..

[62] Yu Z, Duan X, Luo L, Dai S, Ding Z, Xia G (2020). How plant hormones mediate salt stress responses. Trends Plant Sci.

[63] Vurukonda SSKP, Vardharajula S, Shrivastava M, SkZ A (2016). Enhancement of drought stress tolerance in crops by plant growth promoting rhizobacteria. Microbiol Res.

[64] Chen D, Ma X, Li C, Zhang W, Xia G, Wang M (2014). A wheat aminocyclopropane-1-carboxylate oxidase gene, *TaACO1*, negatively regulates salinity stress in *Arabidopsis thaliana*. Plant Cell Rep..

[65] Zhang H, Liu XL, Zhang R-X, Yuan HY, Wang MM, Yang HY, Ma HY, Liu D, Jiang CJ, Liang ZW. Root damage under alkaline stress is associated with reactive oxygen species accumulation in rice (*Oryza sativa* L.). Front Plant Sci. 2017;8:1580. 10.3389/fpls.2017.01580. 10.3389/fpls.2017.01580 PMC5596797 28943882

[66] Yang Q, Zhao D, Zhang C, Wu H, Li Q, Gu M, Sun S, Liu Q (2018). A connection between lysine and serotonin metabolism in rice endosperm. Plant Physiol.

[67] Fang S, Hou X, Liang X (2021). Response mechanisms of plants under saline-alkali stress. Front Plant Sci.

[68] Kaur G, Asthir B (2015). Proline: a key player in plant abiotic stress tolerance. Biol Plant.

[69] Khodabin G, Tahmasebi Sarvestani Z, Rad AHS, Modarres Sanavy SAM (2020). Effect of drought stress on certain morphological and physiological characteristics of a resistant and a sensitive canola cultivar. Chem Biodivers.

[70] Zhu T, Liang C, Meng Z, Sun G, Meng Z, Guo S, Zhang R (2017). CottonFGD: an integrated functional genomics database for cotton. BMC Plant Biol.

[71] Kumar S, Stecher G, Tamura K (2016). MEGA7: molecular evolutionary genetics analysis version 7.0 for bigger datasets. Mol Biol Evol.

[72] Gu X, Wang Y, Gu J (2002). Age distribution of human gene families shows significant roles of both large-and small-scale duplications in vertebrate evolution. Nat Genet.

[73] Wang Y, Tang H, DeBarry JD, Tan X, Li J, Wang X, Lee TH, Jin H, Marler B, Guo H (2012). MCScanX: a toolkit for detection and evolutionary analysis of gene synteny and collinearity. Nucleic Acids Res.

[74] Wang D, Zhang Y, Zhang Z, Zhu J, Yu J (2010). KaKs_Calculator 20: a toolkit incorporating gamma-series methods and sliding window strategies. Genomics Proteomics Bioinform.

[75] Chen C, Chen H, Zhang Y, Thomas HR, Frank MH, He Y, Xia R (2020). TBtools: an integrative toolkit developed for interactive analyses of big biological data. Mol Plant.

[76] Bailey TL, Boden M, Buske FA, Frith M, Grant CE, Clementi L, Ren J, Li WW, Noble WS (2009). MEME SUITE: tools for motif discovery and searching. Nucleic Acids Res.

[77] Hu B, Jin J, Guo A-Y, Zhang H, Luo J, Gao G (2015). GSDS 2.0: an upgraded gene feature visualization server. Bioinformatics.

[78] Hu Y, Chen J, Fang L, Zhang Z, Ma W, Niu Y, Ju L, Deng J, Zhao T, Lian J (2019). *Gossypium barbadense* and *Gossypium hirsutum* genomes provide insights into the origin and evolution of allotetraploid cotton. Nat Genet..

[79] Zhang B, Chen X, Lu X, Shu N, Wang X, Yang X, Wang S, Wang J, Guo L, Wang D. Transcriptome analysis of *Gossypium hirsutum* L. reveals different mechanisms among NaCl, NaOH and Na_2_CO_3_ stress tolerance. Scientific Rep. 2018;8(1):1–14. 10.1038/s41598-018-31668-z. 10.1038/s41598-018-31668-z PMC6131252 30202076

